# Supplementary material for: Bushehr Elderly Health (BEH) programme: study protocol and design of musculoskeletal system and cognitive function (stage II)
Source: BMJ Open. 2017 Aug 4;7(8):e013606. doi: 10.1136/bmjopen-2016-013606 (PMC5577871; doi:10.1136/bmjopen-2016-013606)
Supplement: Author's manuscript [file bmjopen-2016-013606.draft_revisions.pdf]

Bushehr Elderly Health (BEH) program study protocol and design on Musculoskeletal System and Cognitive Function (Stage II)

Journal:	*BMJ Open*
Manuscript ID	bmjopen-2016-013606
Article Type:	Protocol
Date Submitted by the Author:	24-Jul-2016
Complete List of Authors:	Shafiee, Gita ; Endocrinology and Metabolism Population Sciences Institute, Chronic Diseases Research Center<br>Ostovar, Afshin; Bushehr University of Medical Sciences, The Persian Gulf Tropical Medicine Research Center, The Persian Gulf Biomedical Sciences Research Institute<br>Heshmat, Ramin; Endocrinology and Metabolism Population Sciences Institute, Chronic Diseases Research Center<br>Darabi, Hossein ; Bushehr University of Medical Sciences, Bushehr, The Persian Gulf Tropical Medicine Research Center<br>Sharifi, Farshad; Tehran University of Medical Sciences, Elderly Health Research Center, Endocrinology and Metabolism Population Sciences Institute<br>Raeisi, Alireza ; Bushehr University of Medical Sciences, The Persian Gulf Tropical Medicine Research Center<br>Mehrdad, Neda ; Endocrinology and Metabolism Population Sciences Institute, Tehran University of Medical Sciences, Elderly Health Research Center<br>Shadman, Zhaleh ; Endocrinology and Metabolism Clinical Sciences Institute, Tehran University of Medical Sciences, Diabetes Research Center<br>Razi, Faride ; Endocrinology and Metabolism Clinical Sciences Institute, Tehran University of Medical Sciences, Diabetes Research Center<br>Amini, Mohammad Reza ; Endocrinology and Metabolism Clinical Sciences Institute, Tehran University of Medical Sciences, Endocrinology and Metabolism Research Center<br>Arzaghi, Seyed Masoud; Endocrinology and Metabolism Population Sciences Institute, Tehran University of Medical Sciences, Elderly Health Research Center<br>Aghaei Meybodi, Hamidreza ; Endocrinology and Metabolism Clinical Sciences Institute, Tehran University of Medical Sciences, Osteoporosis Research Center<br>Soltani, Akbar ; Endocrinology and Metabolism Clinical Sciences Institute, Tehran University of Medical Sciences, Endocrinology and Metabolism Research Center<br>Nabipour, Iraj; Bushehr University of Medical Sciences, The Persian Gulf Biomedical Sciences Institute<br>Larijani, Bagher; Univ Tehran Med Sci,
**Primary Subject Heading**:	Epidemiology

Secondary Subject Heading:	Geriatric medicine, Public health
Keywords:	Musculoskeletal System, Cognitive Function, Elderly, protocol

SCHOLARONE™ Manuscripts

For peer review only

Cohort Protocol:

Title:

Bushehr Elderly Health (BEH) program study protocol and design on Musculoskeletal System and Cognitive Function (Stage II)

Gita Shafiee[1], Afshin Ostovar[2], Ramin Heshmat[1], Hossein Darabi[2], Farshad Sharifi[3], Alireza Raeisi[2], Neda Mehrdad[3], Zhaleh Shadman[4], Faride Razi[4], Mohammad Reza Amini[5], Seyed Masoud Arzaghi[3], Hamidreza Aghaei Meybodi[6], Akbar Soltani[5], Iraj Nabipour [*7], Bagher Larijani [*5].

1. Chronic Diseases Research Center, Endocrinology and Metabolism Population Sciences Institute, Tehran University of Medical Sciences, Tehran, Iran
2. The Persian Gulf Tropical Medicine Research Center, Bushehr University of Medical Sciences, Bushehr, Iran
3. Elderly Health Research Center, Endocrinology and Metabolism Population Sciences Institute, Tehran University of Medical Sciences, Tehran, Iran
4. Diabetes Research Center, Endocrinology and Metabolism Clinical Sciences Institute, Tehran University of Medical Sciences, Tehran, Iran
5. Endocrinology and Metabolism Research Center, Endocrinology and Metabolism Clinical Sciences Institute, Tehran University of Medical Sciences, Tehran, Iran
6. Osteoporosis Research Center, Endocrinology and Metabolism Clinical Sciences Institute, Tehran University of Medical Sciences, Tehran, Iran
7. The Persian Gulf Marine Biotechnology Research Center, The Persian Gulf Biomedical Sciences Research Institute, Bushehr University of Medical Sciences, Bushehr, Iran

Corresponding Authors:

Bagher Larijani

professor of Endocrinology, EMRC, Dr shariati hospital, north karegar st, Tehran 14114, Iran. E-mail: emrc@tums.ac.ir

Iraj Nabipour

professor of Endocrinology, Gulf Marine Biotechnology Research Center, Bushehr, Iran.

E-mail: inabipour@gmail.com

For peer review only

Abstract:

Introduction:

Musculoskeletal disorders and cognitive diseases are prevalent and they are significant determinants of morbidity and mortality in older adults. The aim of this study is to investigate the prevalence of musculoskeletal and cognitive diseases and their risk factors and also to assess their associations during future follow ups.

Methods and analysis:

Bushehr Elderly Health (BEH) program is a population-based prospective cohort studyis being conducted in Bushehr, a southern province of Iran. The second stage has began from October 2015 and all subjects aged ≥60 years from stage I will be re-invited after 2.5 years. Data including demographic status, lifestyle factors, general health & medical history, mental & functional health, and medication use are collected through a questionnaire. Anthropometric measures, performance tests & muscle strength, blood pressure, and body composition measurements are done. A total 25 cc venous blood is taken, and sera was separated and stored at –80°C for possible future analyses.

Ethics and dissemination:

The study protocol was approved by the ethics committee of Endocrinology and Metabolism Research Institute, affiliatd to Tehran University of Medical Science as well as the Research Ethics Committee of Bushehr University of Medical Sciences. A written informed consent was signed by all the participants. The study findings will show the prevalence of musculoskeletal disease, cognitive impairement, and their risk factors in an elderly population. The associations will be repeated every 5 years, and the participants will be followed during the study to measure the occurrence outcomes. This study will also have the potential to inform the development of beneficial interventions to improve the management of musculoskeletal and cognitive impairement in Iran and other countries in the Middle East.

Our findings will be disseminated via scientific publication as well as presentation to stakeholders including the patients, clinicians, the public and policymakers, via appropriate avenues.

For peer review only

Key words: Musculoskeletal System , Cognitive Function, Elderly, protocol

Introduction

The world population is aging at an unprecedented rate[1]. As in most other countries,the proportion of those 60 years and older in Iran was about 8.2% in 2011 and is expected to rise to 10% in 2020[2]. The rise in the number of older people highlights the surging need for the understanding the impacts and consequences of this population gowth as well as the necessity to conduct research on developing prevention and management strategies for non-communicable diseases. Also, to avoid socioeconomic burden and medical costs, more new effective programs should be explored. Therefore, health and independence of elderly have become one of the main priorities of public health systems[3].In addition, disability is also emerging as an important factor in public health in the world. Many co-morbidities occurring with aging have a great effect on disability. Musculoskeletal disorders are prevalent and they are significant determinants of morbidity and mortality in older adults. Osteoporosis, characterized by low bone mass and micro-architectural deterioration of bone structure, should be considered as the most common bone disease. This disease may lead to an increase risk of fragility fractures at the wrist, hip, and spine[4].Considering the increasing life expectancy, the prevalence of osteoporosis is rising dramatically.According to the International Osteoporosis Foundation estimation, currently 200 million women from different parts of the world suffer from the disabling disease[5]. A meta-analysis study have revealed a high prevalence of osteoporosis (12% of males and 19% of females) and osteopenia (33% of males and 40% of females) in Iranian population[6], adding that some two million of them are at-risk of experiencing osteoporosis-related fracture[7].

Another musculoskeletal disorder is sarcopenia, the age-related decline in muscle mass and function[8]. Decline of muscle mass is started after the age of 50 and an annual loss of muscle mass has been reported to be approximately %1-2[9]. Sarcopenia is a major risk factor of falling, functional limitation and disability in the elderly[10]. Functional impairment and physical disability in Sarcopenic people are 2 to 3 times more likely[11]. Approximately one third of American women and two third of American men over 60 years suffer from Sarcopenia[12].Disability and loss of independence, falls and fractures are the important complications of osteoporosis and sarcopenia. Therefore, researchers regard such conditions as “dysmobility syndrome”[13]. In addition, other diseases such as metabolic syndrome, cardiovascular and respiratory diseases are

For peer review only

associated with sarcopenia[14, 15]. So, the economic burden of sarcopenia is very heavy. Health care costs attributable to sarcopenia in the United States (US) in 2000 were estimated at 18.5 billion dollars[11].

Other important age-related health problems are dementia and cognitive disorders. The incidence of dementia rises with ageand can impose a great health and social burden on aging people.It is estimated that 35.6 million people are living with dementia now and that the number will reach 115.4 million in 2050. In 2010, 57.7% of people with dementia live in developing countries and this is predicted to increase to 70.5% by 2050[16].Becausedementia is a major cause of disability, the early identification of signs of dementia disorders is important. In addition, its significanc is likely to increase more than ever keeping in mind that more-effective medical treatments are designed and developed to slow the disease process. A subjective memory assessment could potentially be informative of early signs of these diseases, because memory is known to be affected in preclinical dementia and Alzheimer's diseases. A subjective memory impairments have consistently been related to depressive symptoms[17, 18].Nevertheless, studies have indicated that subjective memoryimpairment is predictive of incident dementia[19]. The risk factors of cognitive impairment and their effect sizes were not recognized yet. On the other hand, in the eastern Mediterranean region, there are few prospective studis that have assessed the risk factors of neurocognitive disorders.

Depression is the most frequent mental health problem in the elder population and by the year 2020 is reached the second place in DALY's ranking[20]. It is a risk factor for other diseases like demenia and hospitalization [21].The prevalence of depression in later life is uncertain, which partly is due to the lack of diagnostic criteria for the elderly. WHO estimates that the prevalence of this disorder in the elderly population varies between 10% and 20% depending on cultural situations [22].

Given the importance of geriatric diseases, several elderly cohort studies are being conducted in developed countries[23-25]. In the Middle East, there are a few large longitudinal studies with elderly populations[26]. Bushehr Elderly Health (BEH) program is a population-based prospective cohort studyis being conducted in Bushehr, a southern province of Iran. The main aim of its first stage, was to investigate the prevalence of cardiovascular risk factors and their association to major adverse cardiovascular outcomes[27]. In this stage, the study has been designed and implemented to determine the prevalence of musculoskeletal disorders, cognitive impairment

For peer review only

and their risk factors, as well as investigation of falling, fractures, dementia, poor mobility and functional dependence in each follow- up assessment. Also, we will investigate a number of possible molecular mechanisms linking to geriatric diseases.

Bushehr Elderly Health (BEH) program

Study design

The methodology of the BEH program is previously described elsewhere[27], and here, its summary and the methods related to the current phase is explaind. The BEH program is a prospective cohort study aimed at investigating the prevalence of NCD and its associated risk factors. Baseline measurements of first stage was implemented from March 2013 to October 2014. The second stage has began from October 2015 and data recollection is designed to be in 2.5-year intervals.

Four objectives relating to musculoskeletal disorders and cognitive impairment are identified as follows:

- To determine the prevalence dementia and cognitive impairment and their risk factors among elderly Iranians and assessment of associations during future follow ups.
- To estimate the prevalence musculoskeletal disorders (osteoporosis,sarcopenia) and the risk factors among elderly people and assessment of associations during future follow ups.
- To establish a uniform database for performing subsequent studies of the natural history of musculoskeletal disorders and cognitive impairment for planning and evaluating interventions.
- To collect the DNA resource for supporting genetic studies that have sought genes associated with musculoskeletal disorders and cognitive impairment

The study also aims to act as a core resource and provides aframework to support specific sub-studies abouthealthy aging at the national and international level.

Study population

For peer review only

The target population of this cohort study was all participants aged 60 years residing in the cityof Bushehr, the center of a southern province of Iran. Based on the information available from the Bushehr District Health Centre, this population was estimated to be around 10 000. A total of 3000 individuals were selected using multistage cluster random sampling method in the first stage of sampling 2.5 years ago. Details of inclusion and exclusion criteria have been described previously[27] and figure 1 is shown the flowchart of enrolments in stage II.

Outcome measurements

Every participant will be followed up for any medical event (Fracture, Falling, death) annually by telephone call. He/she is asked for any medical conditions by a trained nurse and, if necessary, a trained physician will investigate their hospital records. In the case of mortality, data are collected from hospital or death certificate by local physician and are verified by death registry system in th district health center. the collected data are then evaluated by an outcome committee consisted of principal investigator, internist, endocrinologist, epidemiologist and physician who collected outcome data.

Data collection

In order to collect the necessary data, a questionnaire was developed by expert groups and was tested in a pilot study to identify problems with the parts and to estimate their time of administration.The majority of questions were taken from previousvalidated questionnaires[28-31].

All participants invited to the BEH Program unit were refered to trained nurses, after signing informed written consent,. The participants are interviewed by trained nurses to complete a 162-items questionnaire regarding socio-demographic data, lifestyle factors, general health and medical history, mental and functional health, and medication use.Table 1 presents the parts of the core questionnaire.

Physical activity level in 24 h of sports, work and leisure time on an average weekday is assessed using a valid self-report questionnaire[31]. The intensity of every specific activity is expressed in metabolic equivalents (METs), which allows for the estimation of energy expenditure of specific physical activities[32].

For peer review only

Dietary assessment is performed by a 24-hour dietary recall to obtain detailed information about all foods and beverages consumed in the past 24 hours. The total amount of each specific food and beverage consumed is captured by expert nutritionist. Standard reference tables are used to convert household portions to grams. Data are entered into the nutritionist IV package, modified for Iranian foods to obtain daily energy, nutrient intakes and servings of foods consumed. For mixed dishes, food groups and nutrients are calculated according to their ingredients.

Also, for evaluating the risk of malnutrition in elderly people, we use the Mini Nutritional Assessment (MNA). This practical assessment tool contains: anthropometric measurements, global assessment, dietary questionnaire, and a subjective assessment which enables a subject to be categorized as normal (adequate nutrition), borderline (at risk of malnutrition), or under-nutrition[33].

Mental and functional health assessments are performed by using the mini–mental state examination (MMSE), Mini- Cog, functional assessment staging test (FAST), the 12-Item Short Form Health Survey (SF-12), the patient health questionnaire-9 (PHQ-9) ,Activities of daily living (ADL) and Instrumental activities of daily living(IADL)for all participants. They have already been validated and translated for use in primary care in Iran[28, 29, 34, 35]:

- **Mini Mental State Examination (MMSE)**: A brief quantitative tool that can be used to assess mental status in adults. It is sensitive to screen for cognitive impairment, to estimate the severity of disease, to identify early changes of cognitive status, and to evaluate response to treatment. this tool is an 11-question measure that tests five areas of cognitive function, including orientation, registration, attention and calculation, recall, and language[36].
- **Mini-Cog test**: Another brief screen test for cognitive impairment is the Mini-Cog. It takes approximately 3 minutes to administer. Its components, including 3-item recall and clock drawingare combined to discriminate dementia. It has minimal language content, which reduces cultural and educational bias[37].
- **Functional Assessment Staging Test (FAST)**: The evaluation of changes in functional performance and activities of daily living skills in elderly individuals, especially patient with dementia disease, is performed by FAST. The FAST scale is comprised of 7 major

levels of functioning (from normal adult to severe Cognitive Decline); levels 6 and 7 are additionally divided into sub-stages (11 total)[38].

- **Patient Health Questionnaire (PHQ):** It is a self-report version of the Primary Care Evaluation of Mental Disorders (*PRIME-MD*) diagnostic instrument for common mental disorders [39]. The PHQ-9 has the potential of being a dual-purpose instrument that, with the same 9 items, can establish depressive disorder diagnoses as well as grade depressive symptom severity [40].
- **Short Form Health Survey (SF-12):**A measure of perceived health (health-related quality of life [QoL]) that describes the degree of general physical health status and mental health distress performed by the 12-Item. SF-12 measures 8 health domains, including physical functioning, role physical, bodily pain, general health, vitality, social functioning, role emotional, and mental health. It provides glimpses into mental and physical functioning and overall QOL[41].
- **Activities of Daily Living (ADL) and InstrumentalActivities of Daily Living(IADL)**: They are as part of an older person's functional assessment. ADLs are routine tasks to refer to people's daily self- care activities. ADLs include: eating, bathing, dressing, toileting, transferring (walking) and continence. An individual's ability to perform ADLs is important for determining what type of long-term care (e.g. Nursing-home care or home care) and coverage the individual needs.

IADLs are the complex skills needed to successfully live independently. These skills are cooking, driving, using the telephone or computer, shopping, keeping track of finances and managing medication. Together, ADLs and IADLs represent the skills that usually make people able to manage thir own life as independent adults[42].

Clinical examination

(Table 2)

- **Anthropometric measurements**

Anthropometric measurements are taken with shoes removed and the participants wearing light clothing. Height and weight are measured with a fixedstadiometer and a digital scale according to the standard protocol.A flexible, circumference measuring tape is used to measure waist

For peer review only

circumference (WC);at a point midway between the iliac crest and the lowest rib in standing position, hip circumference; at the most part of the hip.

Neck circumference is measured in the midway of the neck, between mid-cervical spine and mid anterior neck, just below), with the subjects standing upright.Upper arm circumference is measured at the midpoint between the olecranon process and the acromion of right arm, as well, forearm circumference mausurement is done from the widest level while the arm is hanging freely at the side.

Mid-thigh circumference is measured at a midpoint between trochanterion (top of the thigh bone, femur) and tibialelaterale (top of the tibia bone) of right thigh.Calf circumferencemeasurementismeasured at the widest level while the participant is standing upright. All measurements are read to the nearest 0.1 cm.

- **Performance-based tests and muscle strength**

To test muscle function, hand grip strength (muscle strength) , 4.57-meter usual gait speed (physical performance) and the Short Physical Performance Battery (SPPB) are measured[43]. Handgrip strength is measured in both hands using a digital dynamometer, and each hand is measured three times. The main hand is first checked, after which participants are allowed to rest for 15 seconds; then, the other hand is checked. A 4.57-meter walk at the subject's normal pace is timed twice, and the time of the faster of the two walks is used for usual gait speed.

SPPB is a group of measures that combines the results of the gait speed, chair stand and balance tests to measure physical performance in older adults. To test "the chair stand" , participants are asked to keep their arms folded across their chest one time. If successful, participants were asked to stand up and sit down five times and are timed from the initial sitting to final standing position. "Balance tests" include full tandem, semi-tendem, and side by side stands. For each stand, the interviewer supports one arm while participants position their feet, asks if they are ready,then releases the support and begins timing. When 10 seconds have elapsed or participants move their feet, the timer is stopped. To test "gait speed", at 2.44-meter usual gait speed ismeasured.

- **Blood pressure measurments**

Two measurements of systolic blood pressure (SBP) and diastolic blood pressure (DBP) are performed using a standardized mercury sphygmomanometer on the right arm after a 15-minute

For peer review only - http://bmjopen.bmj.com/site/about/guidelines.xhtml

rest in a sitting position; the first and fifth Korotkoff sounds are taken as SBP and DBP, respectively. The average of the two measurements are considered as the participant's blood pressure.

- **Body composition measurments**

Body composition for each participant is measured using dual x-ray absorptiometry (DXA, Discovery Wi, Hologic Inc, , USA). This device can measure fat mass, muscle mass, head, trunk, and extremities separately with minimal radiation exposure. According to DXA results, we will calculate the appendicular skeletal muscle mass for each participant as the sum of upper and lower limb muscle mass. Also, the bone mineral density of the lumbar spine (L1-L4) and total hip are measured in a correct position. All scans are performed by a trained operator on the same day. The time which is required for the evaluation of each person will be 20 minutes.

Biochemical measurements

An overnight fasting venous blood sample is obtained for every participant for biochemical measurements. A total of 25 cc of whole blood is collected by a trained nurse. Table 3 presents laboratory tests performed at baseline and their methods of measurement. Sera are also separated and stored at –80°C for possible future analyses.

Statistical analyses

All data will be analysed using SPSS(version 21; SPSS Inc., Chicago, IL, USA) and STATA (Release 12. Statistical Software.Colllege Station, Texas: StataCorp LP). Normality of variables would be assessed using visual inspection of histograms and the measures of skewness and kurtosis as well as Kolmogrov-Smirnov and Shapiro-Wilks tests. Data will be presented as means±standard deviations (SD), medians and interquartile ranges (IQR) for continuously distributed variables and frequencies and percentages of categorical variables. Appropriate parametric and non-parametric statistical methods will be performed for data analyses. Student t-test or analysis of variance (ANOVA) and their corresponding non-parametric methods (Mann-Whitney and Kruskal-Wallis, respectively) will be used to compare continuous variables among the different groups. Pearson and Spearman correlation coefficients will be performed to assess

For peer review only

the relationship between continuous variables. Pearson Chi-square Test will be used to assess the association of categorical variables. Risk ratio, rate ratio, odds ratio and their 95% confidence intervals will be used to show the association of binary outcomes and corresponding risk factors. Survial analysis will be used for time to event data. Survival rate and hazard ratio will be used to compare between groups.Appropriate multivariable analyses (Linear regression, Logistic regression, Poisson regression, Analysis of Covariance (ANCOVA) methods, Cox-Proportional Hazrd models, etc.) will be used for the purpose of investigating associations after controlling for potential confounders. The level of P value ≤ 0.05 will be considered to indicate statistical significance.

Ethics

The study protocol was approved by the ethics committee of Endocrinology and Metabolism Research Institute (EMRI), affiliatd to Tehran University of Medical Science as well as the Research Ethics Committee of Bushehr University of Medical Sciences. A written informed consent was signed by all the participants including the permission to use secondary data such as registration data for health service purposes, before enrollment in the study

Strengths and limitations

There are several strengths of this study. First, this is the cohort study of older people for a long follow-up time. Therefore, this study may assist in determining causal associations between predicted determinants of health and outcomes and could be used to obtain a multifaceted understanding of geriatric health over time. Also, this study enjoys a large sample size of elderly participants to assess musculoskeletal and cognitive diseases. The face-to-face interview method may be useful in getting exact information, especially among the elderly who may not immediately understand or response the questionnaire. Lastly, our multidisciplinary investigative team consists of experts in geriatrics, psychology, endocrinology, epidemiology, genetics that may be helpful in developing a unique research resource for the understanding of geriatric health.

Attrition bias causd by selection bias as well as recall problems of the elderly people from the very first stage may be considered as the limitations of the current study . Long follow-up and case ascertainment problems should also be mentioned as other limitations. Other limitation of

For peer review only

this study is to enter only those participants with adequate physical ability and the exclusion of those elderly living in nursing homes which has led to selection bias.

Conclusion

This study protocol describes the design and methods used in the stage II of BEH program. In this study, we focused on the epidemiological characteristics of geriatric disorders, the relationship between socioeconomic factors and health and quality of life in addition to elucidating biological risk factors of musculoskeletal and cognitive diseases, and assessment of associations during future follow ups in elderly persons in Bushehr, Iran. The follow-up assessments will be carried out every 5 years for three consecutive periods (a total of 15 years of follow-up), in which all assessments will be repeated with comparable methods and modalities. The study findings will not only improve our understanding of disease prevalence in the elderly of our country, but will also have the potential to inform the development of beneficial interventions to improve the management of musculoskeletal diseases and cognitive impairement in Iran and other countries in the Middle East and North Africa rgion (MENA).

Acknowledgements

The authors are grateful to the staff of both research centres at BPUMS and TUMS for their commitment to the study's protocol and objectives. They are also indebted to all participants who accepted the invitation and patiently underwent exhausting measurements and examinations.

Contributors

GS drafted the manuscript, participated in questionnaire development study design and staff training. AO, RH participated in the study design, questionnaire development and conduction and reviewed the manuscript.HD, FS, AR and ZS participated in questionnaire development, study design and staff training,and reviewed the manuscript. NM, MA, HA, AS participated questionnaire development.IN, BL conceived the study,helped draft the manuscript, participated in the study design and conduction

.

Competing interests

None declared.

For peer review only

Refrences:

1. Interesting facts about ageing. Secondary Interesting facts about ageing. . 2012. http://www.who.int/ageing/about/facts/en/index.html.

2. Selected Findings of National Population and Housing Census,.; 2012, p 27. *In: Public relations and International Cooperation (ed). Tehran: Statistical Center of Iran* 2011**:** 27.

3. Hayat SA, Luben R, Keevil VL *et al.* Cohort profile: A prospective cohort study of objective physical and cognitive capability and visual health in an ageing population of men and women in Norfolk (EPIC-Norfolk 3). *Int J Epidemiol* 2014; 43(4)**:** 1063-72.

4. Larijani B HRH, Bonjour JP, Aghai Meybodi HR, Mohajery Tehrani MR. Osteoporosis in Iran, Overview and Management *Iranian J Publ Health* 2007; supp 1**:** 1-13.

5. JA K. WHO Technical Report, University of Sheffield, UK. 2007**:** 66.

6. Irani AD, Poorolajal J, Khalilian A *et al.* Prevalence of osteoporosis in Iran: A meta-analysis. *J Res Med Sci* 2013; 18(9)**:** 759-66.

7. Keshtkar A LB, Satleghi M, Majidian M, Heshmat R. The incidence of osteoporotic hip fracture in Iran, a review. . *Osteoporos Int 21 (Suppl. 1)* 2010.

8. Cruz-Jentoft AJ, Baeyens JP, Bauer JM *et al.* Sarcopenia: European consensus on definition and diagnosis: Report of the European Working Group on Sarcopenia in Older People. *Age Ageing* 2010; 39(4)**:** 412-23.

9. Masanes F, Culla A, Navarro-Gonzalez M *et al.* Prevalence of sarcopenia in healthy community-dwelling elderly in an urban area of Barcelona (Spain). *J Nutr Health Aging* 2012; 16(2)**:** 184-7.

10. Houston DK, Nicklas BJ, Zizza CA. Weighty concerns: the growing prevalence of obesity among older adults. *J Am Diet Assoc* 2009; 109(11)**:** 1886-95.

11. Janssen I, Shepard DS, Katzmarzyk PT *et al.* The healthcare costs of sarcopenia in the United States. *J Am Geriatr Soc* 2004; 52(1)**:** 80-5.

12. Thomas DR. Loss of skeletal muscle mass in aging: examining the relationship of starvation, sarcopenia and cachexia. *Clin Nutr* 2007; 26(4)**:** 389-99.

For peer review only

13. Binkley N, Krueger D, Buehring B. What's in a name revisited: should osteoporosis and sarcopenia be considered components of "dysmobility syndrome?". *Osteoporos Int* 2013; 24(12): 2955-9.

14. Chien MY, Kuo HK, Wu YT. Sarcopenia, cardiopulmonary fitness, and physical disability in community-dwelling elderly people. *Phys Ther* 2010; 90(9): 1277-87.

15. Chin SO, Rhee SY, Chon S *et al.* Sarcopenia is independently associated with cardiovascular disease in older Korean adults: the Korea National Health and Nutrition Examination Survey (KNHANES) from 2009. *PLoS One* 2013; 8(3): e60119.

16. Organization GWH. Dementia: a public health priority. . 2012.

17. Balash Y, Mordechovich M, Shabtai H *et al.* Subjective memory complaints in elders: depression, anxiety, or cognitive decline? *Acta Neurol Scand* 2013; 127(5): 344-50.

18. Reid M, Parkinson L, Gibson R *et al.* Memory complaint questionnaire performed poorly as screening tool: validation against psychometric tests and affective measures. *J Clin Epidemiol* 2012; 65(2): 199-205.

19. Ronnlund M, Sundstrom A, Adolfsson R *et al.* Self-Reported Memory Failures: Associations with Future Dementia in a Population-Based Study with Long-Term Follow-Up. *J Am Geriatr Soc* 2015; 63(9): 1766-73.

20. Reddy MS. Depression: the disorder and the burden. *Indian J Psychol Med* 2010; 32(1): 1-2.

21. Stoppe G. [Depression in old age]. *Bundesgesundheitsblatt Gesundheitsforschung Gesundheitsschutz* 2008; 51(4): 406-10.

22. organization GWh. World Health Report; Mental Health: New understanding New Hope. 2001.

23. Sharma SK, Tobin JD, Brant LJ. Factors affecting attrition in the Baltimore Longitudinal Study of Aging. *Exp Gerontol* 1986; 21(4-5): 329-40.

24. Hofman A, Grobbee DE, de Jong PT *et al.* Determinants of disease and disability in the elderly: the Rotterdam Elderly Study. *Eur J Epidemiol* 1991; 7(4): 403-22.

25. McDowell I, Hill G, Lindsay J. An overview of the Canadian Study of Health and Aging. *Int Psychogeriatr* 2001; 13 Supp 1: 7-18.

For peer review only

26. Hosseini SR, Cumming RG, Kheirkhah F *et al.* Cohort profile: the Amirkola Health and Ageing Project (AHAP). *Int J Epidemiol* 2014; 43(5)**:** 1393-400.

27. Ostovar A, Nabipour I, Larijani B *et al.* Bushehr Elderly Health (BEH) Programme, phase I (cardiovascular system). *BMJ Open* 2015; 5(12)**:** e009597.

28. Hassani Mehraban A, Soltanmohamadi Y, Akbarfahimi M *et al.* Validity and reliability of the persian version of lawton instrumental activities of daily living scale in patients with dementia. *Med J Islam Repub Iran* 2014; 28**:** 25.

29. Barekatain M, Walterfang M, Behdad M *et al.* Validity and reliability of the Persian language version of the neuropsychiatry unit cognitive assessment tool. *Dement Geriatr Cogn Disord* 2010; 29(6)**:** 516-22.

30. Amirkalali B, Sharifi F, Fakhrzadeh H *et al.* Evaluation of the Mini Nutritional Assessment in the elderly, Tehran, Iran. *Public Health Nutr* 2010; 13(9)**:** 1373-9.

31. Kelishadi R RK, Khosravi AR, Famuri F, Sadeghi M. Assessment of Physical Activity of Adolescents in Isfahan. *J Shahrekord Univ Med Sci* 2001; 3(2)**:** 27-33.

32. Aadahl M, Jorgensen T. Validation of a new self-report instrument for measuring physical activity. *Med Sci Sports Exerc* 2003; 35(7)**:** 1196-202.

33. Vellas B, Guigoz Y, Garry PJ *et al.* The Mini Nutritional Assessment (MNA) and its use in grading the nutritional state of elderly patients. *Nutrition* 1999; 15(2)**:** 116-22.

34. Javadi PS, Zendehbad A, Darabi F *et al.* Development and implementation of Persian test of Elderly for Assessment of Cognition and Executive function (PEACE). *Electron Physician* 2015; 7(7)**:** 1549-56.

35. Montazeri A, Vahdaninia M, Mousavi SJ *et al.* The Iranian version of 12-item Short Form Health Survey (SF-12): factor structure, internal consistency and construct validity. *BMC Public Health* 2009; 9**:** 341.

36. Galea M, Woodward M. Mini-Mental State Examination (MMSE). *Aust J Physiother* 2005; 51(3)**:** 198.

37. Borson S, Scanlan JM, Chen P *et al.* The Mini-Cog as a screen for dementia: validation in a population-based sample. *J Am Geriatr Soc* 2003; 51(10)**:** 1451-4.

For peer review only

38. Sclan SG, Reisberg B. Functional assessment staging (FAST) in Alzheimer's disease: reliability, validity, and ordinality. *Int Psychogeriatr* 1992; 4 Suppl 1**:** 55-69.

39. Spitzer RL, Kroenke K, Linzer M *et al.* Health-related quality of life in primary care patients with mental disorders. Results from the PRIME-MD 1000 Study. *JAMA* 1995; 274(19)**:** 1511-7.

40. Kroenke K, Spitzer RL, Williams JB. The PHQ-9: validity of a brief depression severity measure. *J Gen Intern Med* 2001; 16(9)**:** 606-13.

41. Ware J, Jr., Kosinski M, Keller SD. A 12-Item Short-Form Health Survey: construction of scales and preliminary tests of reliability and validity. *Med Care* 1996; 34(3)**:** 220-33.

42. Katz S. Assessing self-maintenance: activities of daily living, mobility, and instrumental activities of daily living. *J Am Geriatr Soc* 1983; 31(12)**:** 721-7.

43. Guralnik JM, Simonsick EM, Ferrucci L *et al.* A short physical performance battery assessing lower extremity function: association with self-reported disability and prediction of mortality and nursing home admission. *J Gerontol* 1994; 49(2)**:** M85-94.

For peer review only

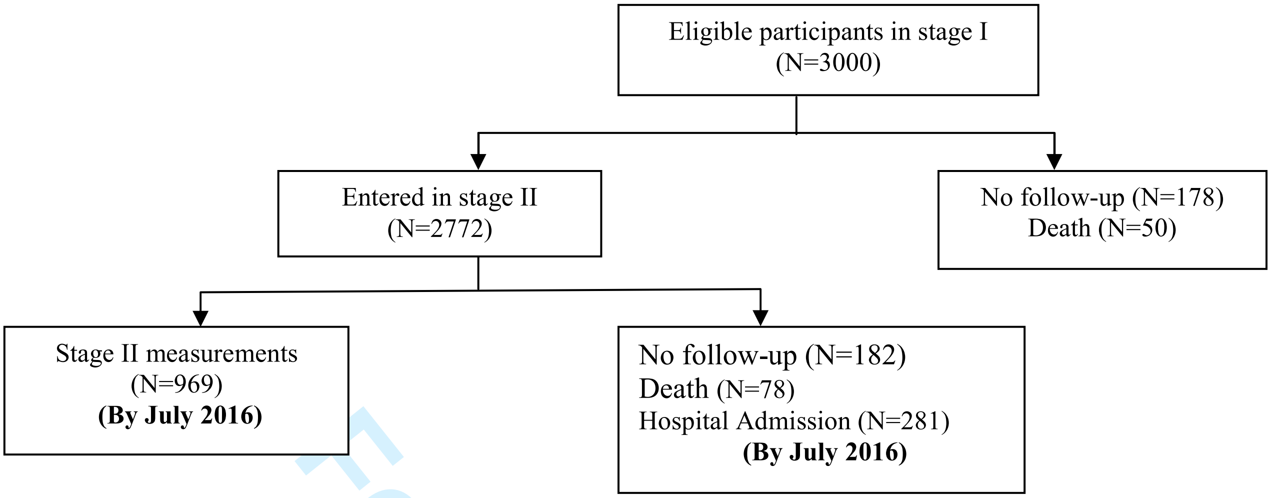

Figure 1. Flow chart of enrolments Stage II

For peer review only

Table 1. Section and topics of the questionnaire in the Bushehr Elderly Health Program study (Stage II)

	Type of data	Parts
1	**Demographic Data**	Personal information
		National identification number
		Age/Sex
		Marital status
		Contact information
2	**Lifestyle factors**	Smoking (current and history)
		Alcohol consumption
		Drug abuse
		Physical activity
		Nutritional assessment
3	**General Health & Medical history**	Quality of life
		Medical history
		Musculoskeletal status (current and history)
		Women's Health
4	**Mental & Functional Health**	Cognitive Function
		Depression
		Functional assessment
		Activities of Daily Living
5	**Medication use**	Administrated by physician
		Over the counter drugs
		Supplements

For peer review only

Table 2. Physical examinations in Bushehr Elderly Health Program study (Stage II)	
• **Anthropometric measures**	
Height and weight	Upper arm circumference
Waist circumference	Forearm circumference
Hip circumference	Mid-thigh circumference
Neck circumference	Calf circumference
• **Performance-based tests**	
1. Short Physical Performance Battery (SPPB)	
➢ Time to perform 5 chair-stands	
➢ Walking 2.44 meters at usual gait speed	
➢ Balance test:	
❖ Side by side standing position	
❖ Full tandem position	
❖ Semi-tendem position	
2. Walking 4.57 meters at usual gait speed	
• **Muscle strength**	
Grip strength	
• **Blood pressure measurments**	
• **Body composition measurments**	
Fat mass	
Muscle mass	
Bone mineral density	

For peer review only

Table 3. Laboratory tests of Bushehr Elderly Health Program study (Phase II)

Laboratory tests	Method of measurement
CBC	Automated hematologyanalyser, Medonic CA620 (Menarini Diagnostics Srl, Florence, Italy)
Fasting blood sugar	Enzymes (glucose oxidase) colorimetric method using a commercial kit (Pars AzmunInc, Karaj, Iran)
Lipid profile	
Total cholesterol	Enzymatic (CHOD-PAP) colorimetric method using a commercial kit (Pars AzmunInc, Karaj, Iran)
LDL-C	Enzymatic (CHE& CHO) colorimetric method using a commercial kit (Pars AzmunInc, Karaj, Iran)
HDL-C	Enzymatic (CHE& CHO) colorimetric method using a commercial kit (Pars AzmunInc, Karaj, Iran)
Triglycerides	Enzymatic (GPO-PAP) colorimetric method using a commercial kit (Pars AzmunInc, Karaj, Iran)
Calcium	Enzymatic colorimetric estimation with o-cresolphthaleincomplexmethod using a commercial kit (Pars AzmunInc,Karaj, Iran)
phosphorus	Enzymatic UV method with phosphomolybdate using a commercial kit (Pars AzmunInc, Karaj, Iran)
ALK-p	Photometric method using a commercial kit (Pars AzmunInc, Karaj, Iran)
Urea	Enzymatic (urease) UVmethod using a commercial kit (Pars AzmunInc, Karaj, Iran)
Creatinin	Jaffe's method, using a Commercial Kit (Pars AzmunInc, Karaj, Iran)
Uric Acid	Colorimetry and uricase method using uric acid TOOS kit (Pars AzmunInc, Karaj, Iran)
HbA1C	Boranate affinity method using a CERA-STAT system (*CERAGEM MEDISYS*, Korea)

CBC, complete blood count; HDL, high-density lipoprotein; LDL, low-density lipoprotein; ALK-p,Alkalan phosphatase; HbA1C, Hemoglobin A1C
CHOD-PAP(cholesterol oxidase phenol aminoantipyrine), CHE(cholesterol esterase), CHO (cholesterol oxidase). GPO-PAP(glycerol-3-phosphateOxidase phenol aminoantipyrine),

For peer review only

Bushehr Elderly Health (BEH) program study protocol and design of Musculoskeletal System and Cognitive Function (Stage II)

Journal:	*BMJ Open*
Manuscript ID	bmjopen-2016-013606.R1
Article Type:	Protocol
Date Submitted by the Author:	07-Dec-2016
Complete List of Authors:	Shafiee, Gita ; Endocrinology and Metabolism Population Sciences Institute, Chronic Diseases Research Center<br>Ostovar, Afshin; Bushehr University of Medical Sciences, The Persian Gulf Tropical Medicine Research Center, The Persian Gulf Biomedical Sciences Research Institute<br>Heshmat, Ramin; Endocrinology and Metabolism Population Sciences Institute, Chronic Diseases Research Center<br>Darabi, Hossein ; Bushehr University of Medical Sciences, Bushehr, The Persian Gulf Tropical Medicine Research Center<br>Sharifi, Farshad; Tehran University of Medical Sciences, Elderly Health Research Center, Endocrinology and Metabolism Population Sciences Institute<br>Raeisi, Alireza ; Bushehr University of Medical Sciences, The Persian Gulf Tropical Medicine Research Center<br>Mehrdad, Neda ; Endocrinology and Metabolism Population Sciences Institute, Tehran University of Medical Sciences, Elderly Health Research Center<br>Shadman, Zhaleh ; Endocrinology and Metabolism Clinical Sciences Institute, Tehran University of Medical Sciences, Diabetes Research Center<br>Razi, Faride ; Endocrinology and Metabolism Clinical Sciences Institute, Tehran University of Medical Sciences, Diabetes Research Center<br>Amini, Mohammad Reza ; Endocrinology and Metabolism Clinical Sciences Institute, Tehran University of Medical Sciences, Endocrinology and Metabolism Research Center<br>Arzaghi, Seyed Masoud; Endocrinology and Metabolism Population Sciences Institute, Tehran University of Medical Sciences, Elderly Health Research Center<br>Aghaei Meybodi, Hamidreza ; Endocrinology and Metabolism Clinical Sciences Institute, Tehran University of Medical Sciences, Osteoporosis Research Center<br>Soltani, Akbar ; Endocrinology and Metabolism Clinical Sciences Institute, Tehran University of Medical Sciences, Endocrinology and Metabolism Research Center<br>Nabipour, Iraj; Bushehr University of Medical Sciences, The Persian Gulf Biomedical Sciences Institute<br>Larijani, Bagher; Univ Tehran Med Sci,
**Primary Subject Heading**:	Epidemiology

For peer review only - http://bmjopen.bmj.com/site/about/guidelines.xhtml

Secondary Subject Heading:	Geriatric medicine, Public health
Keywords:	Musculoskeletal System, Cognitive Function, Elderly, protocol

SCHOLARONE™ Manuscripts

For peer review only

Cohort Protocol:

Title:

Bushehr Elderly Health (BEH) program study protocol and design of Musculoskeletal System and Cognitive Function (Stage II)

Gita Shafiee[1], Afshin Ostovar[2], Ramin Heshmat[1], Hossein Darabi[2], Farshad Sharifi[3], Alireza Raeisi[2], Neda Mehrdad[3], Zhaleh Shadman[4], Faride Razi[4], Mohammad Reza Amini[5],Hamidreza Aghaei Meybodi[6], Akbar Soltani[5], Iraj Nabipour[*7], Bagher Larijani[*5].

1. Chronic Diseases Research Center, Endocrinology and Metabolism Population Sciences Institute, Tehran University of Medical Sciences, Tehran, Iran
2. The Persian Gulf Tropical Medicine Research Center, Bushehr University of Medical Sciences, Bushehr, Iran
3. Elderly Health Research Center, Endocrinology and Metabolism Population Sciences Institute, Tehran University of Medical Sciences, Tehran, Iran
4. Diabetes Research Center, Endocrinology and Metabolism Clinical Sciences Institute, Tehran University of Medical Sciences, Tehran, Iran
5. Endocrinology and Metabolism Research Center, Endocrinology and Metabolism Clinical Sciences Institute, Tehran University of Medical Sciences, Tehran, Iran
6. Osteoporosis Research Center, Endocrinology and Metabolism Clinical Sciences Institute, Tehran University of Medical Sciences, Tehran, Iran
7. The Persian Gulf Marine Biotechnology Research Center, The Persian Gulf Biomedical Sciences Research Institute, Bushehr University of Medical Sciences, Bushehr, Iran

Corresponding Authors:

BagherLarijani

*professor of Endocrinology, EMRC, Drshariati hospital, north karegarst, Tehran 14114, Iran.*E-mail:emrc@tums.ac.ir

IrajNabipour

*professor of Endocrinology,*The Persian Gulf Biomedical sciences Research Institute, Bushehr, Iran.

E-mail:inabipour@gmail.com

For peer review only

Abstract:

Introduction:

Musculoskeletal disorders and cognitive diseases are prevalent and they are significant determinants of morbidity and mortality in older adults. The aim of this study is to investigate the prevalence of musculoskeletal and cognitive diseases and their risk factors and also to assess their associations during future follow ups.

Methods and analysis:

Bushehr Elderly Health (BEH) program is a population-based prospective cohort study being conducted in Bushehr, a southern province of Iran. A total of 3000 older people aged ≥60 were participated in the first stage from which 2772 were eligible to participate in the second stage started after 2.5 years. Data including demographic status, lifestyle factors, general health & medical history, and mental & functional health are collected through a questionnaire. Anthropometric measures, performance tests & muscle strength, blood pressure, and body composition measurements are done. A total 25 cc venous blood is taken, and sera are stored at –80°C for possible future analyses.

Ethics and dissemination:

The study protocol was approved by the ethics committee of Endocrinology and Metabolism Research Institute, affiliated to Tehran University of Medical Science as well as the Research Ethics Committee of Bushehr University of Medical Sciences. A written informed consent was signed by all the participants. The study findings will show the prevalence of musculoskeletal disease, cognitive impairment, and their risk factors in an elderly population. The participants will be followed during the study to measure the occurrence outcomes.

This study will also have the potential to inform the development of beneficial interventions to improve the management of musculoskeletal and cognitive impairment in Iran and other countries in the Middle East.

Our findings will be disseminated via scientific publication as well as presentation to stakeholders, including the patients, clinicians, the public and policymakers, via appropriate avenues.

Key words: Musculoskeletal System, Cognitive Function, Elderly, protocol

For peer review only

Introduction

The world population is aging at an unprecedented rate (1). As in most other countries, the proportion of those 60 years and older in Iran was about 8.2% in 2011 and is expected to rise to 10% in 2020 (2). The rise in the number of older people highlights the surging need for understanding the impacts and consequences of this population growth as well as the necessity to conduct research on developing prevention and management strategies for non-communicable diseases. Also, to avoid the socioeconomic burden and medical costs, more new effective programs should be explored. Therefore, health and independence of the elderly have become one of the main priorities of public health systems (3). In addition, disability is also emerging as an important factor in public health in the world. Many co-morbidities occurring with aging have a great effect on disability. Musculoskeletal disorders are prevalent and they are significant determinants of morbidity and mortality in older adults. Osteoporosis, characterized by low bone mass and micro-architectural deterioration of bone structure, should be considered as the most common bone disease. This disease may lead to an increase risk of fragility fractures at the wrist, hip, and spine (4). Considering the increasing life expectancy, the prevalence of osteoporosis is rising dramatically. According to the International Osteoporosis Foundation estimation, currently 200 million women from different parts of the world suffer from the disabling disease (5). A meta-analysis study have revealed a high prevalence of osteoporosis (12% of males and 19% of females) and osteopenia (33% of males and 40% of females) in Iranian population (6), adding that some two million of them are at-risk of experiencing osteoporosis-related fracture (7).

Another musculoskeletal disorder is sarcopenia, the age-related decline in muscle mass and function (8). Decline of muscle mass is started after the age of 50 and an annual loss of muscle mass has been reported to be approximately %1-2 (9). Sarcopenia is a major risk factor of falling, functional limitation and disability in the elderly (10). Functional impairment and physical disability in sarcopenic people are 2 to 3 times more likely (11). Approximately one third of American women and two third of American men over 60 years suffer from sarcopenia (12). Disability and loss of independence, falls and fractures are the important complications of osteoporosis and sarcopenia. Therefore, researchers regard such conditions as "dysmobility syndrome"(13). In addition, other diseases such as metabolic syndrome, cardiovascular and respiratory diseases are associated with sarcopenia (14, 15). So, the economic burden of

For peer review only

sarcopenia is very heavy. Health care costs attributable to sarcopenia in the United States (US) in 2000 were estimated at 18.5 billion dollars (11).

Other important age-related health problems are dementia and cognitive disorders. The incidence of dementia rises with age and can impose a great health and social burden on aging people. It is estimated that 35.6 million people are living with dementia now and that the number will reach 115.4 million in 2050. In 2010, 57.7% of people with dementia live in developing countries and this is predicted to increase to 70.5% by 2050 (16). Because dementia is a major cause of disability, it is very important to identify signs and symptoms of dementia at the early stages in order to reduce risk of progressing to an advanced disease and impaired cognitive health. In addition, its significance is likely to increase more than ever keeping in mind that more-effective medical treatments are designed and developed to slow the disease process. A subjective memory assessment could potentially be informative of early signs of these diseases, because memory is known to be affected in preclinical dementia and Alzheimer's diseases. Subjective memory impairments have consistently been related to depressive symptoms (17, 18). Nevertheless, studies have indicated that subjective memory impairment is predictive of incident dementia (19). The risk factors of cognitive impairment and their effect sizes were not recognized yet. On the other hand, in the eastern Mediterranean region, there are few prospective studies that have assessed the risk factors of neurocognitive disorders.

Depression is the most frequent mental health problem in the elder population and by the year 2020 has reached the second place in DALY's ranking (20). It is a risk factor for other diseases like dementia and hospitalization (21). The prevalence of depression in later life is uncertain, which partly is due to the lack of diagnostic criteria for the elderly. WHO estimates that the prevalence of this disorder in the elderly population varies between 10% and 20% depending on cultural situations (22).

Given the importance of geriatric diseases, several elderly cohort studies are being conducted in developed countries (23-25). In the Middle East, there are a few large longitudinal studies with elderly populations (26). Bushehr Elderly Health (BEH) program is a population-based prospective cohort study is being conducted in Bushehr, a southern province of Iran. The main aim of its first stage, was to investigate the prevalence of cardiovascular risk factors and their association to major adverse cardiovascular outcomes (27). In this stage, the study has been designed and implemented to determine the prevalence of musculoskeletal disorders, cognitive

For peer review only

impairment and their risk factors, as well as investigation of falling, fractures, dementia, poor mobility and functional dependence in each follow- up assessment. Also, we will investigate a number of possible molecular mechanisms linking to geriatric diseases.

Bushehr Elderly Health (BEH) program

Study design

The methodology of the BEH program is previously described elsewhere (27), and here, its summary and the methods related to the current phase is explained. The BEH program is a prospective cohort study aimed at investigating the prevalence of non- communicable diseases (NCD) and its associated risk factors. Baseline measurements of the first stage were implemented from March 2013 to October 2014. The second stage has begun from October 2015 and data recollection is designed to be in 2.5-year intervals.

Four objectives relating to musculoskeletal disorders and cognitive impairment are identified as follows:

- To determine the prevalence dementia and cognitive impairment and their risk factors among elderly Iranians and assessment of associations with their outcomes during future follow ups.
- To estimate the prevalence musculoskeletal disorders (osteoporosis, sarcopenia) and the risk factors among elderly people and assessment of associations with their outcomes during future follow ups.
- To establish a uniform database for performing subsequent studies of the natural history of musculoskeletal disorders and cognitive impairment for planning and evaluating interventions.
- To collect the DNA resource for supporting genetic studies that have sought genes associated with musculoskeletal disorders and cognitive impairment

The study also aims to act as a core resource and provides a framework to support specific sub-studies about healthy aging at the national and international level.

Study population

For peer review only

The target population of this cohort study was all participants aged 60 years residing in the city of Bushehr, the center of a southern province of Iran. Based on the information available from the Bushehr District Health Centre, this population was estimated to be around 10 000. A total of 3000 individuals were selected using a multistage cluster random sampling method in the first stage of sampling 2.5 years prior to the beginning of the second stage.

This sample size is enough for estimation of dementia prevalence with a rate of 8% (6.5% – 9%) and with a calculated power of 83% STATA (Release 12; Statistical software. College Station, Texas: Stata Corp LP) (28).

The participants had been selected from 75 neighborhoods defined by the municipality. The number of participants recruited from each neighborhood was proportional to the number of households reported from the last census (about 2 years prior to the stage I). The participation rate for the first stage was about 91%. The participants and an accompanied person (usually a relative) signed the informed consent. None of those who came to the recruiting center declined to sign the form.

Older people who were in care homes were excluded from the study. Details of inclusion and exclusion criteria have been described previously (27) and figure 1 shows the flow chart of enrolments in stage II.

Outcome measurements

Every participant will be followed up for any medical event (Fracture, Falling, death) annually by telephone call. He/she has been asked for any medical conditions by a trained nurse and, if necessary, a trained physician will investigate their hospital records. In the case of mortality, data are collected from hospital or death certificate by local physicians and are verified by death registry system in the district health centre. The collected data are then evaluated by an outcome committee consisted of principal investigator, internist, endocrinologist, epidemiologist and physician who collected outcome data.

Data collection

For peer review only

In order to collect the necessary data, a questionnaire was developed by expert groups and was tested in a pilot study to identify problems with the parts and to estimate their time of administration. The majority of questions were taken from previous validated questionnaires (29-33).

All participants invited to the BEH Program unit were referred to trained nurses, after signing informed written consent,. The participants are interviewed by trained nurses to complete a162-items questionnaire regarding socio-demographic data, lifestyle factors, general health and medical history, mental and functional health, and medication use. Table 1 presents the parts of the core questionnaire.

Physical activity level in 24 h of sports, work and leisure time on an average weekday is assessed using a valid self-report questionnaire (33). The intensity of every specific activity is expressed in metabolic equivalents (METs), which allows for the estimation of energy expenditure of specific physical activities (34).

Dietary assessment is performed by a 24-hour dietary recall to obtain detailed information about all foods and beverages consumed in the past 24 hours. The total amount of each specific food and beverage consumed is captured by expert nutritionist. Standard reference tables are used to convert household portions to grams. Data are entered into the nutritionist IV package, modified for Iranian foods to obtain daily energy, nutrient intakes and servings of foods consumed. For mixed dishes, food groups and nutrients are calculated according to their ingredients.

Also, for evaluating the risk of malnutrition in elderly people, we use the Mini Nutritional Assessment (MNA). This practical assessment tool contains: anthropometric measurements, global assessment, dietary questionnaire, and a subjective assessment which enables a subject to be categorized as normal (adequate nutrition), borderline (at risk of malnutrition), or under-nutrition (35).

Mental and functional health assessments are performed by using the mini–mental state examination (MMSE), Mini- Cog, functional assessment staging test (FAST), the 12-Item Short Form Health Survey (SF-12), the patient health questionnaire-9 (PHQ-9), Activities of daily living (ADL) and Instrumental activities of daily living (IADL) for all participants. They have already been validated and translated for use in primary care in Iran (29, 30, 36, 37):

For peer review only

- **Mini Mental State Examination (MMSE)**: A brief quantitative tool that can be used to assess mental status in adults. It is sensitive to screen for cognitive impairment, to estimate the severity of disease, to identify early changes of cognitive status, and to evaluate response to treatment. This tool is an 11-question measure that tests five areas of cognitive function, including orientation, registration, attention and calculation, recall, and language (38).
- **Mini-Cog test**: Another brief screen test for cognitive impairment is the Mini-Cog. It takes approximately 3 minutes to administer. Its components, including 3-item recall and clock drawing are combined to discriminate dementia. It has minimal language content, which reduces cultural and educational bias (39).
- **Functional Assessment Staging Test (FAST)**: The evaluation of changes in functional performance and activities of daily living skills in elderly individuals, especially patient with dementia disease, is performed by FAST. The FAST scale is comprised of 7 major levels of functioning (from normal adult to severe Cognitive Decline); levels 6 and 7 are additionally divided into sub-stages (11 total) (40).
- **Patient Health Questionnaire (PHQ):** It is a self-report version of the Primary Care Evaluation of Mental Disorders (*PRIME-MD*) diagnostic instrument for common mental disorders (41). The PHQ-9 has the potential of being a dual-purpose instrument that, with the same 9 items, can establish depressive disorder diagnoses as well as grade depressive symptom severity (42).
- **Short Form Health Survey (SF-12):** A measure of perceived health (health-related quality of life [QoL]) that describes the degree of general physical health status and mental health distress performed by the 12-Item. SF-12 measures 8 health domains, including physical functioning, role physical, bodily pain, general health, vitality, social functioning, role emotional and mental health. It provides glimpses into mental and physical functioning and overall QOL (43).
- **Activities of Daily Living (ADL) and Instrumental Activities of Daily Living (IADL)**: They are as part of an older person's functional assessment. ADLs are routine tasks to refer to people's daily self- care activities. ADLs include: eating, bathing, dressing, toileting, transferring (walking) and continence. An individual's ability to perform ADLs

For peer review only

is important for determining what type of long-term care (e.g. Nursing-home care or home care) and coverage the individual needs.

IADLs are the complex skills needed to successfully live independently. These skills are cooking, driving, using the telephone or computer, shopping, keeping track of finances and managing medication. Together, ADLs and IADLs represent the skills that usually make people able to manage their own life as independent adults (44).

Clinical examination

(Table 2)

- **Anthropometric measurements**

Anthropometric measurements are taken with shoes removed and the participants wearing light clothing. Height and weight are measured with a fixed stadiometer and a digital scale according to the standard protocol. A flexible, circumference measuring tape is used to measure waist circumference (WC); at a point midway between the iliac crest and the lowest rib in standing position, hip circumference; at the most part of the hip.

Neck circumference is measured in the midway of the neck, between mid-cervical spine and mid anterior neck, just below), with the subjects standing upright. Upper arm circumference is measured at the midpoint between the olecranon process and the acromion of right arm, as well as, forearm circumference measurement is done from the widest level while the arm is hanging freely at the side.

Mid-thigh circumference is measured at a midpoint between trochanterion (top of the thigh bone, femur) and tibiale laterale (top of the tibia bone) of right thigh. Calf circumference measurement is measured at the widest level while the participant is standing upright. All measurements are read to the nearest 0.1 cm.

- **Performance-based tests and muscle strength**

To test muscle function, hand grip strength (muscle strength), 4.57-meter usual gait speed (physical performance) and the Short Physical Performance Battery (SPPB) are measured (45). Handgrip strength is measured in both hands using a digital dynamometer, and each hand is measured three times. The main hand is first checked, after which participants are allowed to rest

For peer review only - http://bmjopen.bmj.com/site/about/guidelines.xhtml

for 15 seconds; then, the other hand is checked. A 4.57-meter walk at the subject's normal pace is timed twice, and the time of the faster of the two walks is used for usual gait speed.

SPPB is a group of measures that combines the results of the gait speed, chair stand and balance tests to measure physical performance in older adults. To test "the chair stand", participants are asked to keep their arms folded across their chest one time. If successful, participants were asked to stand up and sit down five times and are timed from the initial sitting to final standing position. "Balance tests" include full tandem, semi-tandem, and side by side stands. For each stand, the interviewer supports one arm while participants position their feet, asks if they are ready, then releases the support and begins timing. When 10 seconds have elapsed or participants move their feet, the timer is stopped. To test "gait speed", at 2.44-meter usual gait speed is measured.

- **Blood pressure measurements**

Two measurements of systolic blood pressure (SBP) and diastolic blood pressure (DBP) are performed using a standardized mercury sphygmomanometer on the right arm after a 15-minute rest in a sitting position; the first and fifth Korotkoff sounds are taken as SBP and DBP, respectively. The average of the two measurements is considered as the participant's blood pressure.

- **Body composition measurements**

Body composition for each participant is measured using dual x-ray absorptiometry (DXA, Discovery WI, Hologic Inc, USA). This device can measure fat mass, muscle mass, head, trunk, and extremities separately with minimal radiation exposure. According to DXA results, we will calculate the appendicular skeletal muscle mass for each participant as the sum of upper and lower limb muscle mass. Also, the bone mineral density of the lumbar spine (L1-L4) and total hip are measured in a correct position. All scans are performed by a trained operator on the same day. The time which is required for the evaluation of each person will be 20 minutes.

Biochemical measurements

An overnight fasting venous blood sample is obtained for every participant for biochemical measurements. A total of 25 cc of whole blood is collected by a trained nurse. Table 3 presents

For peer review only

laboratory tests performed at baseline and their methods of measurement. Sera are also separated and stored at –80°C for possible future analyses.

DNA will also be extracted from whole peripheral blood stored in EDTA tubes using standard methods for future Genome-, and Epigenome-Wide Association Studies (GWAS and EWAS).

Statistical analyses

All data will be analyzed using SPSS (version 21; SPSS Inc., Chicago, IL, USA) and STATA (Release 12; Statistical Software. College Station, Texas: Stata Corp LP). Normality of variables would be assessed using visual inspection of histograms and the measures of skewness and kurtosis as well as Kolmogrov-Smirnov and Shapiro-Wilks tests. Data will be presented as means± standard deviations (SD), medians and interquartile ranges (IQR) for continuously distributed variables and frequencies and percentages of categorical variables. Point estimation will be standardized using national reference population to be comparable with other studies.

Appropriate parametric and non-parametric statistical methods will be performed for data analyses. Student t- test or analysis of variance (ANOVA) and their corresponding non-parametric methods (Mann-Whitney and Kruskal-Wallis, respectively) will be used to compare continuous variables between the different groups. Pearson and Spearman correlation coefficients will be performed to assess the relationship between continuous variables. Pearson Chi-square Test will be used to assess the association of categorical variables. Risk ratio, rate ratio, odds ratio and their 95% confidence intervals will be used to show the association of binary outcomes and corresponding risk factors. Survival analysis will be used for time to event data. The survival rate and hazard ratio will be used to compare between groups. Appropriate multivariable analyses (Linear regression, Logistic regression, Poisson regression, Analysis of Covariance (ANCOVA) methods, Cox-Proportional Hazard models, etc.) will be used for the purpose of investigating associations after controlling for potential confounders.

Missing data will be treated by performing appropriate methods, including replacing missing values with the mean of the observed values, the last measured values, sensitivity analysis of the best and worst scenarios, and multiple imputation analyses (46).

The level of P value ≤ 0.05 will be considered to indicate statistical significance.

For peer review only

Ethics

The study protocol was approved by the ethics committee of Endocrinology and Metabolism Research Institute (EMRI), affiliated to Tehran University of Medical Science as well as the Research Ethics Committee of Bushehr University of Medical Sciences. A written in forming consent was signed by all the participants, including the permission to use secondary data such as registration data for health service purposes, before enrollment in the study

Strengths and limitations

There are several strengths of this study. First, this is the cohort study of older people for a longer follow-up time. Therefore, this study may assist in determining causal associations between predicted determinants of health and outcomes and could be used to obtain a multifaceted understanding of geriatric health over time. Also, this study enjoys a large sample size of elderly participants to assess musculoskeletal and cognitive diseases. The face-to-face interview method may be useful in getting exact information, especially among the elderly who may not immediately understand or response the questionnaire. Lastly, our multidisciplinary investigative team consists of experts in geriatrics, psychology, endocrinology, epidemiology, genetics that may be helpful in developing a unique research resource for the understanding of geriatric health.

Attrition bias caused by selection bias as well as recall problems of the elderly people from the very first stage may be considered as the limitations of the current study. Long follow-up and case ascertainment problems should also be mentioned as other limitations. Another limitation of this study is to enter only those participants with adequate physical ability and the exclusion of those elderly living in nursing homes which has led to selection bias.

Conclusion

This study protocol describes the design and methods used in the stage II of BEH program. In this study, we focused on the epidemiological characteristics of geriatric disorders, the relationship between socioeconomic factors and health and quality of life in addition to elucidating biological risk factors of musculoskeletal and cognitive diseases, and assessment of associations during future follow ups in elderly persons in Bushehr, Iran. The follow-up assessments for each stage will be carried out every 5 years for three consecutive periods (a total

For peer review only

of 15 years of follow-up), in which all assessments will be repeated with comparable methods and modalities.

The study findings will not only improve our understanding of disease prevalence in the elderly of our country, but will also have the potential to inform the development of beneficial interventions to improve the management of musculoskeletal diseases and cognitive impairment in Iran and other countries in the Middle East and North Africa region (MENA).

Acknowledgements

The authors are grateful to the staff of both research centers at BPUMS and TUMS for their commitment to the study's protocol and objectives. They are also indebted to all participants who accepted the invitation and patiently underwent exhausting measurements and examinations.

Contributors

GS drafted the manuscript, participated in the questionnaire development study design and staff training. AO, RH participated in the study design, questionnaire development and conduction and reviewed the manuscript. HD, FS, AR and ZS participated in questionnaire development, study design and staff training, and reviewed the manuscript. NM, MA, HA, AS participated questionnaire development. IN, BL conceived the study, helped draft the manuscript, participated in the study design and conduction

Data sharing statement

Data will be available for interested researchers upon their request via our website (http://www.elderly.bpums.ac.ir) or through direct email to the executive manager AO (a.ostovar@bpums.ac.ir; afshinostovar@gmail.com).

.

Competing interests

None declared.

For peer review only

References:

1. Interesting facts about ageing. Secondary Interesting facts about ageing. . 2012. http://www.who.int/ageing/about/facts/en/index.html.
2. Selected Findings of National Population and Housing Census,.; 2012, p 27. In: Public relations and International Cooperation (ed) Tehran: Statistical Center of Iran. 2011:27.
3. Hayat SA, Luben R, Keevil VL, Moore S, Dalzell N, Bhaniani A, et al. Cohort profile: A prospective cohort study of objective physical and cognitive capability and visual health in an ageing population of men and women in Norfolk (EPIC-Norfolk 3). Int J Epidemiol. 2014;43(4):1063-72.
4. Larijani B HRH, Bonjour JP, Aghai Meybodi HR, Mohajery Tehrani MR. Osteoporosis in Iran, Overview and Management Iranian J Publ Health. 2007;supp 1:1-13.
5. JA K. WHO Technical Report, University of Sheffield, UK. 2007:66.
6. Irani AD, Poorolajal J, Khalilian A, Esmailnasab N, Cheraghi Z. Prevalence of osteoporosis in Iran: A meta-analysis. J Res Med Sci. 2013;18(9):759-66.
7. Keshtkar A LB, Satleghi M, Majidian M, Heshmat R The incidence of osteoporotic hip fracture in Iran, a review. . Osteoporos Int 21 (Suppl 1). 2010.
8. Cruz-Jentoft AJ, Baeyens JP, Bauer JM, Boirie Y, Cederholm T, Landi F, et al. Sarcopenia: European consensus on definition and diagnosis: Report of the European Working Group on Sarcopenia in Older People. Age Ageing. 2010;39(4):412-23.
9. Masanes F, Culla A, Navarro-Gonzalez M, Navarro-Lopez M, Sacanella E, Torres B, et al. Prevalence of sarcopenia in healthy community-dwelling elderly in an urban area of Barcelona (Spain). J Nutr Health Aging. 2012;16(2):184-7.
10. Houston DK, Nicklas BJ, Zizza CA. Weighty concerns: the growing prevalence of obesity among older adults. J Am Diet Assoc. 2009;109(11):1886-95.
11. Janssen I, Shepard DS, Katzmarzyk PT, Roubenoff R. The healthcare costs of sarcopenia in the United States. J Am Geriatr Soc. 2004;52(1):80-5.
12. Thomas DR. Loss of skeletal muscle mass in aging: examining the relationship of starvation, sarcopenia and cachexia. Clin Nutr. 2007;26(4):389-99.
13. Binkley N, Krueger D, Buehring B. What's in a name revisited: should osteoporosis and sarcopenia be considered components of "dysmobility syndrome?". Osteoporos Int. 2013;24(12):2955-9.
14. Chien MY, Kuo HK, Wu YT. Sarcopenia, cardiopulmonary fitness, and physical disability in community-dwelling elderly people. Phys Ther. 2010;90(9):1277-87.
15. Chin SO, Rhee SY, Chon S, Hwang YC, Jeong IK, Oh S, et al. Sarcopenia is independently associated with cardiovascular disease in older Korean adults: the Korea National Health and Nutrition Examination Survey (KNHANES) from 2009. PLoS One. 2013;8(3):e60119.
16. Organization GWH. Dementia: a public health priority. . 2012.
17. Balash Y, Mordechovich M, Shabtai H, Giladi N, Gurevich T, Korczyn AD. Subjective memory complaints in elders: depression, anxiety, or cognitive decline? Acta Neurol Scand. 2013;127(5):344-50.
18. Reid M, Parkinson L, Gibson R, Schofield P, D'Este C, Attia J, et al. Memory complaint questionnaire performed poorly as screening tool: validation against psychometric tests and affective measures. J Clin Epidemiol. 2012;65(2):199-205.
19. Ronnlund M, Sundstrom A, Adolfsson R, Nilsson LG. Self-Reported Memory Failures: Associations with Future Dementia in a Population-Based Study with Long-Term Follow-Up. J Am Geriatr Soc. 2015;63(9):1766-73.
20. Reddy MS. Depression: the disorder and the burden. Indian J Psychol Med. 2010;32(1):1-2.

For peer review only

21. Stoppe G. [Depression in old age]. Bundesgesundheitsblatt Gesundheitsforschung Gesundheitsschutz. 2008;51(4):406-10.
22. organization GWh. World Health Report; Mental Health: New understanding New Hope. 2001.
23. Sharma SK, Tobin JD, Brant LJ. Factors affecting attrition in the Baltimore Longitudinal Study of Aging. Exp Gerontol. 1986;21(4-5):329-40.
24. Hofman A, Grobbee DE, de Jong PT, van den Ouweland FA. Determinants of disease and disability in the elderly: the Rotterdam Elderly Study. Eur J Epidemiol. 1991;7(4):403-22.
25. McDowell I, Hill G, Lindsay J. An overview of the Canadian Study of Health and Aging. Int Psychogeriatr. 2001;13 Supp 1:7-18.
26. Hosseini SR, Cumming RG, Kheirkhah F, Nooreddini H, Baiani M, Mikaniki E, et al. Cohort profile: the Amirkola Health and Ageing Project (AHAP). Int J Epidemiol. 2014;43(5):1393-400.
27. Ostovar A, Nabipour I, Larijani B, Heshmat R, Darabi H, Vahdat K, et al. Bushehr Elderly Health (BEH) Programme, phase I (cardiovascular system). BMJ Open. 2015;5(12):e009597.
28. Sharifi F FH, Varmaghani M, et al. Prevalence of Dementia and Associated Factors among Older Adults in Iran: National Elderly Health Survey (NEHS). Arch Iran Medin press. 2016.
29. Hassani Mehraban A, Soltanmohamadi Y, Akbarfahimi M, Taghizadeh G. Validity and reliability of the persian version of lawton instrumental activities of daily living scale in patients with dementia. Med J Islam Repub Iran. 2014;28:25.
30. Barekatain M, Walterfang M, Behdad M, Tavakkoli M, Mahvari J, Maracy MR, et al. Validity and reliability of the Persian language version of the neuropsychiatry unit cognitive assessment tool. Dement Geriatr Cogn Disord. 2010;29(6):516-22.
31. Amirkalali B, Sharifi F, Fakhrzadeh H, Mirarefin M, Ghaderpanahi M, Larijani B. Evaluation of the Mini Nutritional Assessment in the elderly, Tehran, Iran. Public Health Nutr. 2010;13(9):1373-9.
32. Kelishadi R RK, Khosravi AR, Famuri F, Sadeghi M. Assessment of Physical Activity of Adolescents in Isfahan. J Shahrekord Univ Med Sci. 2001;3(2):27-33.
33. Ara I, Aparicio-Ugarriza R, Morales-Barco D, Nascimento de Souza W, Mata E, Gonzalez-Gross M. Physical activity assessment in the general population; validated self-report methods. Nutr Hosp. 2015;31 Suppl 3:211-8.
34. Aadahl M, Jorgensen T. Validation of a new self-report instrument for measuring physical activity. Med Sci Sports Exerc. 2003;35(7):1196-202.
35. Vellas B, Guigoz Y, Garry PJ, Nourhashemi F, Bennahum D, Lauque S, et al. The Mini Nutritional Assessment (MNA) and its use in grading the nutritional state of elderly patients. Nutrition. 1999;15(2):116-22.
36. Javadi PS, Zendehbad A, Darabi F, Khosravifar S, Noroozian M. Development and implementation of Persian test of Elderly for Assessment of Cognition and Executive function (PEACE). Electron Physician. 2015;7(7):1549-56.
37. Montazeri A, Vahdaninia M, Mousavi SJ, Omidvari S. The Iranian version of 12-item Short Form Health Survey (SF-12): factor structure, internal consistency and construct validity. BMC Public Health. 2009;9:341.
38. Galea M, Woodward M. Mini-Mental State Examination (MMSE). Aust J Physiother. 2005;51(3):198.
39. Borson S, Scanlan JM, Chen P, Ganguli M. The Mini-Cog as a screen for dementia: validation in a population-based sample. J Am Geriatr Soc. 2003;51(10):1451-4.
40. Sclan SG, Reisberg B. Functional assessment staging (FAST) in Alzheimer's disease: reliability, validity, and ordinality. Int Psychogeriatr. 1992;4 Suppl 1:55-69.
41. Spitzer RL, Kroenke K, Linzer M, Hahn SR, Williams JB, deGruy FV, 3rd, et al. Health-related quality of life in primary care patients with mental disorders. Results from the PRIME-MD 1000 Study. JAMA. 1995;274(19):1511-7.

For peer review only

42. Kroenke K, Spitzer RL, Williams JB. The PHQ-9: validity of a brief depression severity measure. J Gen Intern Med. 2001;16(9):606-13.
43. Ware J, Jr., Kosinski M, Keller SD. A 12-Item Short-Form Health Survey: construction of scales and preliminary tests of reliability and validity. Med Care. 1996;34(3):220-33.
44. Katz S. Assessing self-maintenance: activities of daily living, mobility, and instrumental activities of daily living. J Am Geriatr Soc. 1983;31(12):721-7.
45. Guralnik JM, Simonsick EM, Ferrucci L, Glynn RJ, Berkman LF, Blazer DG, et al. A short physical performance battery assessing lower extremity function: association with self-reported disability and prediction of mortality and nursing home admission. J Gerontol. 1994;49(2):M85-94.
46. Sterne JA, White IR, Carlin JB, Spratt M, Royston P, Kenward MG, et al. Multiple imputation for missing data in epidemiological and clinical research: potential and pitfalls. Bmj. 2009;338:b2393.

For peer review only

Figure legends:

Figure1. Flow chart of enrolments Stage II

For peer review only

Table 1. Section and topics of the questionnaire in the Bushehr Elderly Health Program study (Stage II)

	Type of data	Parts
1	**Demographic Data**	Personal information
		National identification number
		Age/Sex
		Marital status
		Contact information
2	**Lifestyle factors**	Smoking (current and history)
		Alcohol consumption
		Drug abuse
		Physical activity
		Nutritional assessment
3	**General Health & Medical history**	Quality of life
		Medical history
		Musculoskeletal status (current and history)
		Women's Health
4	**Mental & Functional Health**	Cognitive Function
		Depression
		Functional assessment
		Activities of Daily Living
5	**Medication use**	Administrated by physician
		Over the counter drugs
		Supplements

For peer review only

Table 2. Physical examinations in Bushehr Elderly Health Program study (Stage II)

- **Anthropometric measures**
  - Height and weight
  - Waist circumference
  - Hip circumference
  - Neck circumference
  - Upper arm circumference
  - Forearm circumference
  - Mid-thigh circumference
  - Calf circumference
- **Performance-based tests**
  1. Short Physical Performance Battery (SPPB)
     - Time to perform 5 chair-stands
     - Walking 2.44 meters at usual gait speed
     - Balance test:
       - Side by side standing position
       - Full tandem position
       - Semi-tandem position
  2. Walking 4.57 meters at usual gait speed
- **Muscle strength**
  - Grip strength
- **Blood pressure measurements**
- **Body composition measurements**
  - Fat mass
  - Muscle mass
  - Bone mineral density

For peer review only

Table 3. Laboratory tests of Bushehr Elderly Health Program study (Phase II)	
**Laboratory tests**	**Method of measurement**
CBC	Automated hematology analyzer, Medonic CA620 (Menarini Diagnostics Srl, Florence, Italy)
Fasting blood sugar	Enzymes (glucose oxidase) colorimetric method using a commercial kit (Pars Azmun Inc., Karaj, Iran)
Lipid profile	
Total cholesterol	Enzymatic (CHOD-PAP) colorimetric method using a commercial kit (Pars Azmun Inc, Karaj, Iran)
LDL-C	Enzymatic (CHE& CHO) colorimetric method using a commercial kit (Pars Azmun Inc, Karaj, Iran)
HDL-C	Enzymatic (CHE& CHO) colorimetric method using a commercial kit (Pars Azmun Inc, Karaj, Iran)
Triglycerides	Enzymatic (GPO-PAP) colorimetric method using a commercial kit (Pars Azmun Inc, Karaj, Iran)
Calcium	Enzymatic colorimetric estimation with o-cresolphthalein complex method using a commercial kit (Pars Azmun Inc, Karaj, Iran)
phosphorus	Enzymatic UV method with phosphomolybdate using a commercial kit (Pars Azmun Inc, Karaj, Iran)
ALK-p	Photometric method using a commercial kit (Pars Azmun Inc, Karaj, Iran)
Urea	Enzymatic (urease) UV method using a commercial kit (Pars Azmun Inc, Karaj, Iran)
Creatinin	Jaffe's method, using a Commercial Kit (Pars Azmun Inc, Karaj, Iran)
Uric Acid	Colorimetry and uricase method using uric acid TOOS kit (Pars Azmun Inc, Karaj, Iran)
HbA1C	Boranate affinity method using a CERA-STAT system (*CERAGEM MEDISYS*, Korea)

CBC, complete blood count; HDL, high-density lipoprotein; LDL, low-density lipoprotein; ALK-p,Alkalan phosphatase; HbA1C, Hemoglobin A1C
CHOD-PAP (cholesterol oxidase phenol aminoantipyrine), CHE (cholesterol esterase), CHO (cholesterol oxidase). GPO-PAP(glycerol-3-phosphateOxidase phenol aminoantipyrine),

For peer review only

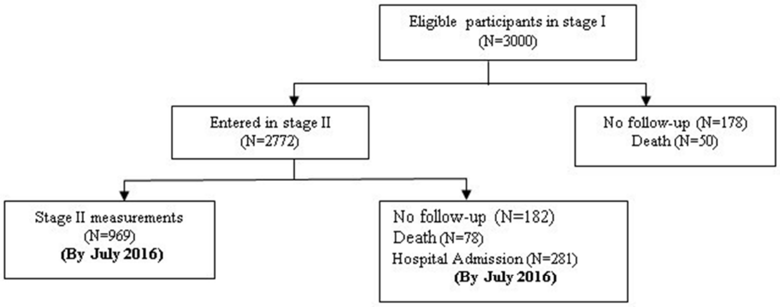

Figure1. Flow chart of enrolments Stage II

216x121mm (96 x 96 DPI)

review only

BMJ Open

Bushehr Elderly Health (BEH) program: Study protocol and design of Musculoskeletal System and Cognitive Function (Stage II)

Journal:	*BMJ Open*
Manuscript ID	bmjopen-2016-013606.R2
Article Type:	Protocol
Date Submitted by the Author:	21-Feb-2017
Complete List of Authors:	Shafiee, Gita ; Endocrinology and Metabolism Population Sciences Institute, Chronic Diseases Research Center<br>Ostovar, Afshin; Bushehr University of Medical Sciences, The Persian Gulf Tropical Medicine Research Center, The Persian Gulf Biomedical Sciences Research Institute<br>Heshmat, Ramin; Endocrinology and Metabolism Population Sciences Institute, Chronic Diseases Research Center<br>Darabi, Hossein ; Bushehr University of Medical Sciences, Bushehr, The Persian Gulf Tropical Medicine Research Center<br>Sharifi, Farshad; Tehran University of Medical Sciences, Elderly Health Research Center, Endocrinology and Metabolism Population Sciences Institute<br>Raeisi, Alireza ; Bushehr University of Medical Sciences, The Persian Gulf Tropical Medicine Research Center<br>Mehrdad, Neda ; Endocrinology and Metabolism Population Sciences Institute, Tehran University of Medical Sciences, Elderly Health Research Center<br>Shadman, Zhaleh ; Endocrinology and Metabolism Clinical Sciences Institute, Tehran University of Medical Sciences, Diabetes Research Center<br>Razi, Faride ; Endocrinology and Metabolism Clinical Sciences Institute, Tehran University of Medical Sciences, Diabetes Research Center<br>Amini, Mohammad Reza ; Endocrinology and Metabolism Clinical Sciences Institute, Tehran University of Medical Sciences, Endocrinology and Metabolism Research Center<br>Arzaghi, Seyed Masoud; Endocrinology and Metabolism Population Sciences Institute, Tehran University of Medical Sciences, Elderly Health Research Center<br>Aghaei Meybodi, Hamidreza ; Endocrinology and Metabolism Clinical Sciences Institute, Tehran University of Medical Sciences, Osteoporosis Research Center<br>Soltani, Akbar ; Endocrinology and Metabolism Clinical Sciences Institute, Tehran University of Medical Sciences, Endocrinology and Metabolism Research Center<br>Nabipour, Iraj; Bushehr University of Medical Sciences, The Persian Gulf Biomedical Sciences Institute<br>Larijani, Bagher; Univ Tehran Med Sci,
**Primary Subject Heading**:	Epidemiology

Secondary Subject Heading:	Geriatric medicine, Public health
Keywords:	Musculoskeletal System, Cognitive Function, Elderly, protocol

SCHOLARONE™ Manuscripts

For peer review only

Cohort Protocol:

Title:

Bushehr Elderly Health (BEH) program: Study protocol and design of Musculoskeletal System and Cognitive Function (Stage II)

Gita Shafiee[1], Afshin Ostovar[2], Ramin Heshmat[1], Hossein Darabi[2], Farshad Sharifi[3], Alireza Raeisi[2], Neda Mehrdad[3], Zhaleh Shadman[4], Faride Razi[4], Mohammad Reza Amini[5],Hamidreza Aghaei Meybodi[6], Akbar Soltani[5], Iraj Nabipour[*7], Bagher Larijani[*5].

1. Chronic Diseases Research Center, Endocrinology and Metabolism Population Sciences Institute, Tehran University of Medical Sciences, Tehran, Iran
2. The Persian Gulf Tropical Medicine Research Center, Bushehr University of Medical Sciences, Bushehr, Iran
3. Elderly Health Research Center, Endocrinology and Metabolism Population Sciences Institute, Tehran University of Medical Sciences, Tehran, Iran
4. Diabetes Research Center, Endocrinology and Metabolism Clinical Sciences Institute, Tehran University of Medical Sciences, Tehran, Iran
5. Endocrinology and Metabolism Research Center, Endocrinology and Metabolism Clinical Sciences Institute, Tehran University of Medical Sciences, Tehran, Iran
6. Osteoporosis Research Center, Endocrinology and Metabolism Clinical Sciences Institute, Tehran University of Medical Sciences, Tehran, Iran
7. The Persian Gulf Marine Biotechnology Research Center, The Persian Gulf Biomedical Sciences Research Institute, Bushehr University of Medical Sciences, Bushehr, Iran

Corresponding Authors:

BagherLarijani

*professor of Endocrinology, EMRC, Drshariati hospital, north karegarst, Tehran 14114, Iran.*E-mail:emrc@tums.ac.ir

IrajNabipour

*professor of Endocrinology,*The Persian Gulf Biomedical sciences Research Institute, Bushehr, Iran.

E-mail:inabipour@gmail.com

For peer review only

Abstract:

Introduction:

Musculoskeletal disorders and cognitive diseases are prevalent and they are significant determinants of morbidity and mortality in older adults. The aim of this study is to investigate the prevalence of musculoskeletal and cognitive diseases and their risk factors and also to assess their associations during future follow ups.

Methods and analysis:

Bushehr Elderly Health (BEH) program is a population-based prospective cohort study being conducted in Bushehr, a southern province of Iran. A total of 3000 older people aged ≥60 were participated in the first stage from which 2772 were eligible to participate in the second stage started after 2.5 years. Data including demographic status, lifestyle factors, general health & medical history, and mental & functional health are collected through a questionnaire. Anthropometric measures, performance tests & muscle strength, blood pressure, and body composition measurements are done. A total 25 cc venous blood is taken, and sera are stored at –80°C for possible future analyses.

Ethics and dissemination:

The study protocol was approved by the ethics committee of Endocrinology and Metabolism Research Institute, affiliated to Tehran University of Medical Science as well as the Research Ethics Committee of Bushehr University of Medical Sciences. A written informed consent was signed by all the participants. The study findings will show the prevalence of musculoskeletal disease, cognitive impairment, and their risk factors in an elderly population. The participants will be followed during the study to measure the occurrence outcomes.

This study will also have the potential to inform the development of beneficial interventions to improve the management of musculoskeletal and cognitive impairment in Iran and other countries in the Middle East.

Our findings will be disseminated via scientific publication as well as presentation to stakeholders, including the patients, clinicians, the public and policymakers, via appropriate avenues.

Key words: Musculoskeletal System, Cognitive Function, Elderly, protocol

For peer review only

Strengths and limitations of this study

- This is the large sample prospective cohort study in Iran focusing on musculoskeletal disorders and cognitive impairment in the elderly as a growing population.
- Future follow-ups in this study will allow for the assessment of many relevant outcomes
- Collection of the DNA resource for supporting genetic studies in this study will be sought genes associated with musculoskeletal disorders and cognitive impairment
- The participation rate in this study is high; however, those participants with inadequate physical ability and those elderly living in nursing homes are excluded

Introduction

The world population is aging at an unprecedented rate (1). As in most other countries, the proportion of those 60 years and older in Iran was about 8.2% in 2011 and is expected to rise to 10% in 2020 (2). The rise in the number of older people highlights the surging need for understanding the impacts and consequences of this population growth as well as the necessity to conduct research on developing prevention and management strategies for non-communicable diseases. Also, to avoid the socioeconomic burden and medical costs, more new effective programs should be explored. Therefore, health and independence of the elderly have become one of the main priorities of public health systems (3). In addition, disability is also emerging as an important factor in public health in the world. Many co-morbidities occurring with aging have a great effect on disability. Musculoskeletal disorders are prevalent and they are significant determinants of morbidity and mortality in older adults. Osteoporosis, characterized by low bone mass and micro-architectural deterioration of bone structure, should be considered as the most common bone disease. This disease may lead to an increase risk of fragility fractures at the wrist, hip, and spine (4). Considering the increasing life expectancy, the prevalence of osteoporosis is rising dramatically. According to the International Osteoporosis Foundation estimation, currently 200 million women from different parts of the world suffer from the disabling disease (5). A meta-analysis study have revealed a high prevalence of osteoporosis (12% of males and 19% of females) and osteopenia (33% of males and 40% of females) in Iranian population (6), adding that some two million of them are at-risk of experiencing osteoporosis-related fracture (7).

For peer review only

Another musculoskeletal disorder is sarcopenia, the age-related decline in muscle mass and function (8). Decline of muscle mass is started after the age of 50 and an annual loss of muscle mass has been reported to be approximately %1-2 (9). Sarcopenia is a major risk factor of falling, functional limitation and disability in the elderly (10). Functional impairment and physical disability in sarcopenic people are 2 to 3 times more likely (11). Approximately one third of American women and two third of American men over 60 years suffer from sarcopenia (12). Disability and loss of independence, falls and fractures are the important complications of osteoporosis and sarcopenia. Therefore, researchers regard such conditions as "dysmobility syndrome"(13). In addition, other diseases such as metabolic syndrome, cardiovascular and respiratory diseases are associated with sarcopenia (14, 15). So, the economic burden of sarcopenia is very heavy. Health care costs attributable to sarcopenia in the United States (US) in 2000 were estimated at 18.5 billion dollars (11).

Other important age-related health problems are dementia and cognitive disorders. The incidence of dementia rises with age and can impose a great health and social burden on aging people. It is estimated that 35.6 million people are living with dementia now and that the number will reach 115.4 million in 2050. In 2010, 57.7% of people with dementia live in developing countries and this is predicted to increase to 70.5% by 2050 (16). Because dementia is a major cause of disability, it is very important to identify signs and symptoms of dementia at the early stages in order to reduce risk of progressing to an advanced disease and impaired cognitive health. In addition, its significance is likely to increase more than ever keeping in mind that more-effective medical treatments are designed and developed to slow the disease process. A subjective memory assessment could potentially be informative of early signs of these diseases, because memory is known to be affected in preclinical dementia and Alzheimer's diseases. Subjective memory impairments have consistently been related to depressive symptoms (17, 18). Nevertheless, studies have indicated that subjective memory impairment is predictive of incident dementia (19). The risk factors of cognitive impairment and their effect sizes were not recognized yet. On the other hand, in the eastern Mediterranean region, there are few prospective studies that have assessed the risk factors of neurocognitive disorders.

Depression is the most frequent mental health problem in the elder population and by the year 2020 has reached the second place in DALY's ranking (20). It is a risk factor for other diseases like dementia and hospitalization (21). The prevalence of depression in later life is uncertain,

For peer review only

which partly is due to the lack of diagnostic criteria for the elderly. WHO estimates that the prevalence of this disorder in the elderly population varies between 10% and 20% depending on cultural situations (22).

Given the importance of geriatric diseases, several elderly cohort studies are being conducted in developed countries (23-25). In the Middle East, there are a few large longitudinal studies with elderly populations (26). Bushehr Elderly Health (BEH) program is a population-based prospective cohort study is being conducted in Bushehr, a southern province of Iran. The main aim of its first stage, was to investigate the prevalence of cardiovascular risk factors and their association to major adverse cardiovascular outcomes (27). In this stage, the study has been designed and implemented to determine the prevalence of musculoskeletal disorders, cognitive impairment and their risk factors, as well as investigation of falling, fractures, dementia, poor mobility and functional dependence in each follow- up assessment. Also, we will investigate a number of possible molecular mechanisms linking to geriatric diseases.

Bushehr Elderly Health (BEH) program

Study design

The methodology of the BEH program is previously described elsewhere (27), and here, its summary and the methods related to the current phase is explained. The BEH program is a prospective cohort study aimed at investigating the prevalence of non- communicable diseases (NCD) and its associated risk factors. Baseline measurements of the first stage were implemented from March 2013 to October 2014. The second stage has begun from October 2015 and data recollection is designed to be in 2.5-year intervals.

Four objectives relating to musculoskeletal disorders and cognitive impairment are identified as follows:

- To determine the prevalence dementia and cognitive impairment and their risk factors among elderly Iranians and assessment of associations with their outcomes during future follow ups.
- To estimate the prevalence musculoskeletal disorders (osteoporosis, sarcopenia) and the risk factors among elderly people and assessment of associations with their outcomes during future follow ups.

For peer review only - http://bmjopen.bmj.com/site/about/guidelines.xhtml

- To establish a uniform database for performing subsequent studies of the natural history of musculoskeletal disorders and cognitive impairment for planning and evaluating interventions.
- To collect the DNA resource for supporting genetic studies that have sought genes associated with musculoskeletal disorders and cognitive impairment

The study also aims to act as a core resource and provides a framework to support specific sub-studies about healthy aging at the national and international level.

Study population

The target population of this cohort study was all participants aged 60 years residing in the city of Bushehr, the center of a southern province of Iran. Based on the information available from the Bushehr District Health Centre, this population was estimated to be around 10 000. A total of 3000 individuals were selected using a multistage cluster random sampling method in the first stage of sampling 2.5 years prior to the beginning of the second stage.

This sample size is enough for estimation of dementia prevalence with a rate of 8% (6.5% – 9%) and with a calculated power of 83% STATA (Release 12; Statistical software. College Station, Texas: Stata Corp LP) (28).

The participants had been selected from 75 neighborhoods defined by the municipality. The number of participants recruited from each neighborhood was proportional to the number of households reported from the last census (about 2 years prior to the stage I). The participation rate for the first stage was about 91%. The participants and an accompanied person (usually a relative) signed the informed consent. None of those who came to the recruiting center declined to sign the form.

Older people who were in care homes were excluded from the study. Details of inclusion and exclusion criteria have been described previously (27) and figure 1 shows the flow chart of enrolments in stage II.

Outcome measurements

For peer review only

Every participant will be followed up for any medical event (Fracture, Falling, death) annually by telephone call. He/she has been asked for any medical conditions by a trained nurse and, if necessary, a trained physician will investigate their hospital records. In the case of mortality, data are collected from hospital or death certificate by local physicians and are verified by death registry system in the district health centre. The collected data are then evaluated by an outcome committee consisted of principal investigator, internist, endocrinologist, epidemiologist and physician who collected outcome data.

Data collection

In order to collect the necessary data, a questionnaire was developed by expert groups and was tested in a pilot study to identify problems with the parts and to estimate their time of administration. The majority of questions were taken from previous validated questionnaires (29-32).

All participants invited to the BEH Program unit were referred to trained nurses, after signing informed written consent. The participants are interviewed by trained nurses to complete a162-items questionnaire regarding socio-demographic data, lifestyle factors, general health and medical history, mental and functional health, and medication use. Table 1 presents the parts of the core questionnaire.

Physical activity level in 24 h of sports, work and leisure time on an average weekday is assessed using a valid self-report questionnaire (33) , and the intensity of every specific activity is expressed in metabolic equivalents (METs). This instrument has been validated and used for elderlies (34-36) and also, it has been validated among Iranian adolescents for Farsi language (32)

Dietary assessment is performed by a 24-hour dietary recall to obtain detailed information about all foods and beverages consumed in the past 24 hours. The total amount of each specific food and beverage consumed is captured by expert nutritionist. Standard reference tables are used to convert household portions to grams. Data are entered into the nutritionist IV package, modified for Iranian foods to obtain daily energy, nutrient intakes and servings of foods consumed. For mixed dishes, food groups and nutrients are calculated according to their ingredients.

For peer review only

Also, for evaluating the risk of malnutrition in elderly people, we use the Mini Nutritional Assessment (MNA). This practical assessment tool contains: anthropometric measurements, global assessment, dietary questionnaire, and a subjective assessment which enables a subject to be categorized as normal (adequate nutrition), borderline (at risk of malnutrition), or under-nutrition (37).

Mental and functional health assessments are performed by using the mini–mental state examination (MMSE), Mini- Cog, functional assessment staging test (FAST), the 12-Item Short Form Health Survey (SF-12), the patient health questionnaire-9 (PHQ-9), Activities of daily living (ADL) and Instrumental activities of daily living (IADL) for all participants. They have already been validated and translated for use in primary care in Iran (29, 30, 38, 39):

- **Mini Mental State Examination (MMSE)**: A brief quantitative tool that can be used to assess mental status in adults. It is sensitive to screen for cognitive impairment, to estimate the severity of disease, to identify early changes of cognitive status, and to evaluate response to treatment. This tool is an 11-question measure that tests five areas of cognitive function, including orientation, registration, attention and calculation, recall, and language (40).
- **Mini-Cog test**: Another brief screen test for cognitive impairment is the Mini-Cog. It takes approximately 3 minutes to administer. Its components, including 3-item recall and clock drawing are combined to discriminate dementia. It has minimal language content, which reduces cultural and educational bias (41).
- **Functional Assessment Staging Test (FAST)**: The evaluation of changes in functional performance and activities of daily living skills in elderly individuals, especially patient with dementia disease, is performed by FAST. The FAST scale is comprised of 7 major levels of functioning (from normal adult to severe Cognitive Decline); levels 6 and 7 are additionally divided into sub-stages (11 total) (42).
- **Patient Health Questionnaire (PHQ):** It is a self-report version of the Primary Care Evaluation of Mental Disorders (*PRIME-MD*) diagnostic instrument for common mental disorders (43). The PHQ-9 has the potential of being a dual-purpose instrument that, with the same 9 items, can establish depressive disorder diagnoses as well as grade depressive symptom severity (44).

For peer review only

- **Short Form Health Survey (SF-12):** A measure of perceived health (health-related quality of life [QoL]) that describes the degree of general physical health status and mental health distress performed by the 12-Item. SF-12 measures 8 health domains, including physical functioning, role physical, bodily pain, general health, vitality, social functioning, role emotional and mental health. It provides glimpses into mental and physical functioning and overall QOL (45).
- **Activities of Daily Living (ADL) and Instrumental Activities of Daily Living (IADL)**: They are as part of an older person's functional assessment. ADLs are routine tasks to refer to people's daily self- care activities. ADLs include: eating, bathing, dressing, toileting, transferring (walking) and continence. An individual's ability to perform ADLs is important for determining what type of long-term care (e.g. Nursing-home care or home care) and coverage the individual needs.

IADLs are the complex skills needed to successfully live independently. These skills are cooking, driving, using the telephone or computer, shopping, keeping track of finances and managing medication. Together, ADLs and IADLs represent the skills that usually make people able to manage their own life as independent adults (46).

Clinical examination

(Table 2)

- **Anthropometric measurements**

Anthropometric measurements are taken with shoes removed and the participants wearing light clothing. Height and weight are measured with a fixed stadiometer and a digital scale according to the standard protocol. A flexible, circumference measuring tape is used to measure waist circumference (WC); at a point midway between the iliac crest and the lowest rib in standing position, hip circumference; at the most part of the hip.

Neck circumference is measured in the midway of the neck, between mid-cervical spine and mid anterior neck, just below), with the subjects standing upright. Upper arm circumference is measured at the midpoint between the olecranon process and the acromion of right arm, as well as, forearm circumference measurement is done from the widest level while the arm is hanging freely at the side.

For peer review only

Mid-thigh circumference is measured at a midpoint between trochanterion (top of the thigh bone, femur) and tibiale laterale (top of the tibia bone) of right thigh. Calf circumference measurement is measured at the widest level while the participant is standing upright. All measurements are read to the nearest 0.1 cm.

- **Performance-based tests and muscle strength**

To test muscle function, hand grip strength (muscle strength), 4.57-meter usual gait speed (physical performance) and the Short Physical Performance Battery (SPPB) are measured (47). Handgrip strength is measured in both hands using a digital dynamometer, and each hand is measured three times. The main hand is first checked, after which participants are allowed to rest for 15 seconds; then, the other hand is checked. A 4.57-meter walk at the subject's normal pace is timed twice, and the time of the faster of the two walks is used for usual gait speed.

SPPB is a group of measures that combines the results of the gait speed, chair stand and balance tests to measure physical performance in older adults. To test "the chair stand", participants are asked to keep their arms folded across their chest one time. If successful, participants were asked to stand up and sit down five times and are timed from the initial sitting to final standing position. "Balance tests" include full tandem, semi-tandem, and side by side stands. For each stand, the interviewer supports one arm while participants position their feet, asks if they are ready, then releases the support and begins timing. When 10 seconds have elapsed or participants move their feet, the timer is stopped. To test "gait speed", at 2.44-meter usual gait speed is measured.

- **Blood pressure measurements**

Two measurements of systolic blood pressure (SBP) and diastolic blood pressure (DBP) are performed using a standardized mercury sphygmomanometer on the right arm after a 15-minute rest in a sitting position; the first and fifth Korotkoff sounds are taken as SBP and DBP, respectively. The average of the two measurements is considered as the participant's blood pressure.

- **Body composition measurements**

Body composition for each participant is measured using dual x-ray absorptiometry (DXA, Discovery WI, Hologic Inc, USA). This device can measure fat mass, muscle mass, head, trunk,

For peer review only

and extremities separately with minimal radiation exposure. According to DXA results, we will calculate the appendicular skeletal muscle mass for each participant as the sum of upper and lower limb muscle mass. Also, the bone mineral density of the lumbar spine (L1-L4) and total hip are measured in a correct position. All scans are performed by a trained operator on the same day. The time which is required for the evaluation of each person will be 20 minutes.

Biochemical measurements

An overnight fasting venous blood sample is obtained for every participant for biochemical measurements. A total of 25 cc of whole blood is collected by a trained nurse. Table 3 presents laboratory tests performed at baseline and their methods of measurement. Sera are also separated and stored at –80°C for possible future analyses.

DNA will also be extracted from whole peripheral blood stored in EDTA tubes using standard methods for future Genome-, and Epigenome-Wide Association Studies (GWAS and EWAS).

Statistical analyses

All data will be analyzed using SPSS (version 21; SPSS Inc., Chicago, IL, USA) and STATA (Release 12; Statistical Software. College Station, Texas: Stata Corp LP). Normality of variables would be assessed using visual inspection of histograms and the measures of skewness and kurtosis as well as Kolmogrov-Smirnov and Shapiro-Wilks tests. Data will be presented as means± standard deviations (SD), medians and interquartile ranges (IQR) for continuously distributed variables and frequencies and percentages of categorical variables. For comparing descriptive results of the population, prevalence or incidence rates will be standardized based on country population at the same age- sex groups.

Appropriate parametric and non-parametric statistical methods will be performed for data analyses. Student t- test or analysis of variance (ANOVA) and their corresponding non-parametric methods (Mann-Whitney and Kruskal-Wallis, respectively) will be used to compare continuous variables between the different groups. Pearson and Spearman correlation coefficients will be performed to assess the relationship between continuous variables. Pearson

For peer review only

Chi-square Test will be used to assess the association of categorical variables. Risk ratio, rate ratio, odds ratio and their 95% confidence intervals will be used to show the association of binary outcomes and corresponding risk factors. Survival analysis will be used for time to event data. The survival rate and hazard ratio will be used to compare between groups. Appropriate multivariable analyses (Linear regression, Logistic regression, Poisson regression, Analysis of Covariance (ANCOVA) methods, Cox-Proportional Hazard models, etc.) will be used for the purpose of investigating associations after controlling for potential confounders.

Missing data will be treated by performing appropriate methods, including replacing missing values with the mean of the observed values, the last measured values, sensitivity analysis of the best and worst scenarios, and multiple imputation analyses (48).

The level of P value ≤ 0.05 will be considered to indicate statistical significance.

Ethics

The study protocol was approved by the ethics committee of Endocrinology and Metabolism Research Institute (EMRI), affiliated to Tehran University of Medical Science as well as the Research Ethics Committee of Bushehr University of Medical Sciences. A written in forming consent was signed by all the participants, including the permission to use secondary data such as registration data for health service purposes, before enrollment in the study

Strengths and limitations

There are several strengths of this study. First, this is the cohort study of older people for a longer follow-up time. Therefore, this study may assist in determining causal associations between predicted determinants of health and outcomes and could be used to obtain a multifaceted understanding of geriatric health over time. Also, this study enjoys a large sample size of elderly participants to assess musculoskeletal and cognitive diseases. The face-to-face interview method may be useful in getting exact information, especially among the elderly who may not immediately understand or response the questionnaire. Lastly, our multidisciplinary investigative team consists of experts in geriatrics, psychology, endocrinology, epidemiology, genetics that may be helpful in developing a unique research resource for the understanding of geriatric health.

For peer review only

Attrition bias caused by selection bias as well as recall problems of the elderly people from the very first stage may be considered as the limitations of the current study. Long follow-up and case ascertainment problems should also be mentioned as other limitations. Another limitation of this study is to enter only those participants with adequate physical ability and the exclusion of those elderly living in nursing homes which has led to selection bias. The self-reported physical activity scale tool has been validated in Farsi language only among Iranian adolescents and not especially in elderlies. However, it has been validated for elderlies in other studies.

Conclusion

This study protocol describes the design and methods used in the stage II of BEH program. In this study, we focused on the epidemiological characteristics of geriatric disorders, the relationship between socioeconomic factors and health and quality of life in addition to elucidating biological risk factors of musculoskeletal and cognitive diseases, and assessment of associations during future follow ups in elderly persons in Bushehr, Iran. The follow-up assessments for each stage will be carried out every 5 years for three consecutive periods (a total of 15 years of follow-up), in which all assessments will be repeated with comparable methods and modalities.

The study findings will not only improve our understanding of disease prevalence in the elderly of our country, but will also have the potential to inform the development of beneficial interventions to improve the management of musculoskeletal diseases and cognitive impairment in Iran and other countries in the Middle East and North Africa region (MENA).

Acknowledgements

The authors are grateful to the staff of both research centers at BPUMS and TUMS for their commitment to the study's protocol and objectives. They are also indebted to all participants who accepted the invitation and patiently underwent exhausting measurements and examinations.

Contributors

GS drafted the manuscript, participated in the questionnaire development study design and staff training. AO, RH participated in the study design, questionnaire development and conduction

For peer review only

and reviewed the manuscript. HD, FS, AR and ZS participated in questionnaire development, study design and staff training, and reviewed the manuscript. NM, MA, HA, AS participated questionnaire development. IN, BL conceived the study, helped draft the manuscript, participated in the study design and conduction

Data sharing statement

Data will be available for interested researchers upon their request via our website (http://www.elderly.bpums.ac.ir) or through direct email to the executive manager AO (a.ostovar@bpums.ac.ir; afshinostovar@gmail.com).

.

Competing interests: None declared.

References:

1. Interesting facts about ageing. Secondary Interesting facts about ageing. . 2012. http://www.who.int/ageing/about/facts/en/index.html.
2. Selected Findings of National Population and Housing Census,.; 2012, p 27. In: Public relations and International Cooperation (ed) Tehran: Statistical Center of Iran. 2011:27.
3. Hayat SA, Luben R, Keevil VL, Moore S, Dalzell N, Bhaniani A, et al. Cohort profile: A prospective cohort study of objective physical and cognitive capability and visual health in an ageing population of men and women in Norfolk (EPIC-Norfolk 3). Int J Epidemiol. 2014;43(4):1063-72. Epub 2013/06/19.
4. Larijani B HRH, Bonjour JP, Aghai Meybodi HR, Mohajery Tehrani MR. Osteoporosis in Iran, Overview and Management Iranian J Publ Health. 2007;supp 1:1-13.
5. JA K. WHO Technical Report, University of Sheffield, UK. 2007:66.
6. Irani AD, Poorolajal J, Khalilian A, Esmailnasab N, Cheraghi Z. Prevalence of osteoporosis in Iran: A meta-analysis. J Res Med Sci. 2013;18(9):759-66. Epub 2014/01/02.
7. Keshtkar A LB, Satleghi M, Majidian M, Heshmat R The incidence of osteoporotic hip fracture in Iran, a review. . Osteoporos Int 21 (Suppl 1). 2010.
8. Cruz-Jentoft AJ, Baeyens JP, Bauer JM, Boirie Y, Cederholm T, Landi F, et al. Sarcopenia: European consensus on definition and diagnosis: Report of the European Working Group on Sarcopenia in Older People. Age Ageing. 2010;39(4):412-23. Epub 2010/04/16.
9. Masanes F, Culla A, Navarro-Gonzalez M, Navarro-Lopez M, Sacanella E, Torres B, et al. Prevalence of sarcopenia in healthy community-dwelling elderly in an urban area of Barcelona (Spain). J Nutr Health Aging. 2012;16(2):184-7. Epub 2012/02/11.
10. Houston DK, Nicklas BJ, Zizza CA. Weighty concerns: the growing prevalence of obesity among older adults. J Am Diet Assoc. 2009;109(11):1886-95. Epub 2009/10/28.
11. Janssen I, Shepard DS, Katzmarzyk PT, Roubenoff R. The healthcare costs of sarcopenia in the United States. J Am Geriatr Soc. 2004;52(1):80-5. Epub 2003/12/23.

For peer review only

12. Thomas DR. Loss of skeletal muscle mass in aging: examining the relationship of starvation, sarcopenia and cachexia. Clin Nutr. 2007;26(4):389-99. Epub 2007/05/15.
13. Binkley N, Krueger D, Buehring B. What's in a name revisited: should osteoporosis and sarcopenia be considered components of "dysmobility syndrome?". Osteoporos Int. 2013;24(12):2955-9. Epub 2013/08/02.
14. Chien MY, Kuo HK, Wu YT. Sarcopenia, cardiopulmonary fitness, and physical disability in community-dwelling elderly people. Phys Ther. 2010;90(9):1277-87. Epub 2010/07/10.
15. Chin SO, Rhee SY, Chon S, Hwang YC, Jeong IK, Oh S, et al. Sarcopenia is independently associated with cardiovascular disease in older Korean adults: the Korea National Health and Nutrition Examination Survey (KNHANES) from 2009. PLoS One. 2013;8(3):e60119. Epub 2013/03/28.
16. Organization GWH. Dementia: a public health priority. . 2012.
17. Balash Y, Mordechovich M, Shabtai H, Giladi N, Gurevich T, Korczyn AD. Subjective memory complaints in elders: depression, anxiety, or cognitive decline? Acta Neurol Scand. 2013;127(5):344-50. Epub 2012/12/12.
18. Reid M, Parkinson L, Gibson R, Schofield P, D'Este C, Attia J, et al. Memory complaint questionnaire performed poorly as screening tool: validation against psychometric tests and affective measures. J Clin Epidemiol. 2012;65(2):199-205. Epub 2011/09/06.
19. Ronnlund M, Sundstrom A, Adolfsson R, Nilsson LG. Self-Reported Memory Failures: Associations with Future Dementia in a Population-Based Study with Long-Term Follow-Up. J Am Geriatr Soc. 2015;63(9):1766-73. Epub 2015/08/19.
20. Reddy MS. Depression: the disorder and the burden. Indian J Psychol Med. 2010;32(1):1-2. Epub 2010/01/01.
21. Stoppe G. [Depression in old age]. Bundesgesundheitsblatt Gesundheitsforschung Gesundheitsschutz. 2008;51(4):406-10. Epub 2008/03/18. Depressionen im Alter.
22. organization GWh. World Health Report; Mental Health: New understanding New Hope. 2001.
23. Sharma SK, Tobin JD, Brant LJ. Factors affecting attrition in the Baltimore Longitudinal Study of Aging. Exp Gerontol. 1986;21(4-5):329-40. Epub 1986/01/01.
24. Hofman A, Grobbee DE, de Jong PT, van den Ouweland FA. Determinants of disease and disability in the elderly: the Rotterdam Elderly Study. Eur J Epidemiol. 1991;7(4):403-22. Epub 1991/07/01.
25. McDowell I, Hill G, Lindsay J. An overview of the Canadian Study of Health and Aging. Int Psychogeriatr. 2001;13 Supp 1:7-18. Epub 2002/03/15.
26. Hosseini SR, Cumming RG, Kheirkhah F, Nooreddini H, Baiani M, Mikaniki E, et al. Cohort profile: the Amirkola Health and Ageing Project (AHAP). Int J Epidemiol. 2014;43(5):1393-400. Epub 2013/08/07.
27. Ostovar A, Nabipour I, Larijani B, Heshmat R, Darabi H, Vahdat K, et al. Bushehr Elderly Health (BEH) Programme, phase I (cardiovascular system). BMJ Open. 2015;5(12):e009597. Epub 2015/12/18.
28. Sharifi F FH, Varmaghani M, et al. Prevalence of Dementia and Associated Factors among Older Adults in Iran: National Elderly Health Survey (NEHS). Arch Iran Medin press. 2016.
29. Hassani Mehraban A, Soltanmohamadi Y, Akbarfahimi M, Taghizadeh G. Validity and reliability of the persian version of lawton instrumental activities of daily living scale in patients with dementia. Med J Islam Repub Iran. 2014;28:25. Epub 2014/09/25.
30. Barekatain M, Walterfang M, Behdad M, Tavakkoli M, Mahvari J, Maracy MR, et al. Validity and reliability of the Persian language version of the neuropsychiatry unit cognitive assessment tool. Dement Geriatr Cogn Disord. 2010;29(6):516-22. Epub 2010/06/05.
31. Amirkalali B, Sharifi F, Fakhrzadeh H, Mirarefin M, Ghaderpanahi M, Larijani B. Evaluation of the Mini Nutritional Assessment in the elderly, Tehran, Iran. Public Health Nutr. 2010;13(9):1373-9. Epub 2010/03/02.

For peer review only

32. Kelishadi R , Famuri F, Sadeghi M. Assessment of Physical Activity of Adolescents in Isfahan. J Shahrekord Univ Med Sci. 2001;3(2):27-33.
33. Aadahl M, Jorgensen T. Validation of a new self-report instrument for measuring physical activity. Med Sci Sports Exerc. 2003;35(7):1196-202. Epub 2003/07/04.
34. Andersen LG, Groenvold M, Jorgensen T, Aadahl M. Construct validity of a revised Physical Activity Scale and testing by cognitive interviewing. Scand J Public Health. 2010;38(7):707-14. Epub 2010/09/09.
35. Aadahl M, Kjaer M, Kristensen JH, Mollerup B, Jorgensen T. Self-reported physical activity compared with maximal oxygen uptake in adults. Eur J Cardiovasc Prev Rehabil. 2007;14(3):422-8. Epub 2007/06/15.
36. Aadahl M, Andreasen AH, Hammer-Helmich L, Buhelt L, Jorgensen T, Glumer C. Recent temporal trends in sleep duration, domain-specific sedentary behaviour and physical activity. A survey among 25-79-year-old Danish adults. Scand J Public Health. 2013;41(7):706-11. Epub 2013/06/27.
37. Vellas B, Guigoz Y, Garry PJ, Nourhashemi F, Bennahum D, Lauque S, et al. The Mini Nutritional Assessment (MNA) and its use in grading the nutritional state of elderly patients. Nutrition. 1999;15(2):116-22. Epub 1999/02/17.
38. Javadi PS, Zendehbad A, Darabi F, Khosravifar S, Noroozian M. Development and implementation of Persian test of Elderly for Assessment of Cognition and Executive function (PEACE). Electron Physician. 2015;7(7):1549-56. Epub 2016/01/15.
39. Montazeri A, Vahdaninia M, Mousavi SJ, Omidvari S. The Iranian version of 12-item Short Form Health Survey (SF-12): factor structure, internal consistency and construct validity. BMC Public Health. 2009;9:341. Epub 2009/09/18.
40. Galea M, Woodward M. Mini-Mental State Examination (MMSE). Aust J Physiother. 2005;51(3):198. Epub 2005/09/29.
41. Borson S, Scanlan JM, Chen P, Ganguli M. The Mini-Cog as a screen for dementia: validation in a population-based sample. J Am Geriatr Soc. 2003;51(10):1451-4. Epub 2003/09/27.
42. Sclan SG, Reisberg B. Functional assessment staging (FAST) in Alzheimer's disease: reliability, validity, and ordinality. Int Psychogeriatr. 1992;4 Suppl 1:55-69. Epub 1992/01/01.
43. Spitzer RL, Kroenke K, Linzer M, Hahn SR, Williams JB, deGruy FV, 3rd, et al. Health-related quality of life in primary care patients with mental disorders. Results from the PRIME-MD 1000 Study. JAMA. 1995;274(19):1511-7. Epub 1995/11/15.
44. Kroenke K, Spitzer RL, Williams JB. The PHQ-9: validity of a brief depression severity measure. J Gen Intern Med. 2001;16(9):606-13. Epub 2001/09/15.
45. Ware J, Jr., Kosinski M, Keller SD. A 12-Item Short-Form Health Survey: construction of scales and preliminary tests of reliability and validity. Med Care. 1996;34(3):220-33. Epub 1996/03/01.
46. Katz S. Assessing self-maintenance: activities of daily living, mobility, and instrumental activities of daily living. J Am Geriatr Soc. 1983;31(12):721-7. Epub 1983/12/01.
47. Guralnik JM, Simonsick EM, Ferrucci L, Glynn RJ, Berkman LF, Blazer DG, et al. A short physical performance battery assessing lower extremity function: association with self-reported disability and prediction of mortality and nursing home admission. J Gerontol. 1994;49(2):M85-94. Epub 1994/03/01.
48. Sterne JA, White IR, Carlin JB, Spratt M, Royston P, Kenward MG, et al. Multiple imputation for missing data in epidemiological and clinical research: potential and pitfalls. Bmj. 2009;338:b2393.

For peer review only

Figure legends:

Figure1. Flow chart of enrolments Stage II

For peer review only

Table 1. Section and topics of the questionnaire in the Bushehr Elderly Health Program study (Stage II)		
	**Type of data**	**Parts**
1	**Demographic Data**	Personal information
		National identification number
		Age/Sex
		Marital status
		Contact information
2	**Lifestyle factors**	Smoking (current and history)
		Alcohol consumption
		Drug abuse
		Physical activity
		Nutritional assessment
3	**General Health & Medical history**	Quality of life
		Medical history
		Musculoskeletal status (current and history)
		Women's Health
4	**Mental & Functional Health**	Cognitive Function
		Depression
		Functional assessment
		Activities of Daily Living
5	**Medication use**	Administrated by physician
		Over the counter drugs
		Supplements

For peer review only

Table 2. Physical examinations in Bushehr Elderly Health Program study (Stage II)	
• **Anthropometric measures**	
Height and weight	Upper arm circumference
Waist circumference	Forearm circumference
Hip circumference	Mid-thigh circumference
Neck circumference	Calf circumference
• **Performance-based tests**	
1. Short Physical Performance Battery (SPPB)	
➢ Time to perform 5 chair-stands	
➢ Walking 2.44 meters at usual gait speed	
➢ Balance test:	
❖ Side by side standing position	
❖ Full tandem position	
❖ Semi-tandem position	
2. Walking 4.57 meters at usual gait speed	
• **Muscle strength**	
Grip strength	
• **Blood pressure measurements**	
• **Body composition measurements**	
Fat mass	
Muscle mass	
Bone mineral density	

For peer review only

Table 3. Laboratory tests of Bushehr Elderly Health Program study (Phase II)	
**Laboratory tests**	**Method of measurement**
CBC	Automated hematology analyzer, Medonic CA620 (Menarini Diagnostics Srl, Florence, Italy)
Fasting blood sugar	Enzymes (glucose oxidase) colorimetric method using a commercial kit (Pars Azmun Inc., Karaj, Iran)
Lipid profile	
Total cholesterol	Enzymatic (CHOD-PAP) colorimetric method using a commercial kit (Pars Azmun Inc, Karaj, Iran)
LDL-C	Enzymatic (CHE& CHO) colorimetric method using a commercial kit (Pars Azmun Inc, Karaj, Iran)
HDL-C	Enzymatic (CHE& CHO) colorimetric method using a commercial kit (Pars Azmun Inc, Karaj, Iran)
Triglycerides	Enzymatic (GPO-PAP) colorimetric method using a commercial kit (Pars Azmun Inc, Karaj, Iran)
Calcium	Enzymatic colorimetric estimation with o-cresolphthalein complex method using a commercial kit (Pars Azmun Inc, Karaj, Iran)
phosphorus	Enzymatic UV method with phosphomolybdate using a commercial kit (Pars Azmun Inc, Karaj, Iran)
ALK-p	Photometric method using a commercial kit (Pars Azmun Inc, Karaj, Iran)
Urea	Enzymatic (urease) UV method using a commercial kit (Pars Azmun Inc, Karaj, Iran)
Creatinin	Jaffe's method, using a Commercial Kit (Pars Azmun Inc, Karaj, Iran)
Uric Acid	Colorimetry and uricase method using uric acid TOOS kit (Pars Azmun Inc, Karaj, Iran)
HbA1C	Boranate affinity method using a CERA-STAT system (*CERAGEM MEDISYS*, Korea)

CBC, complete blood count; HDL, high-density lipoprotein; LDL, low-density lipoprotein; ALK-p,Alkalan phosphatase; HbA1C, Hemoglobin A1C
CHOD-PAP (cholesterol oxidase phenol aminoantipyrine), CHE (cholesterol esterase), CHO (cholesterol oxidase). GPO-PAP(glycerol-3-phosphateOxidase phenol aminoantipyrine),

For peer review only

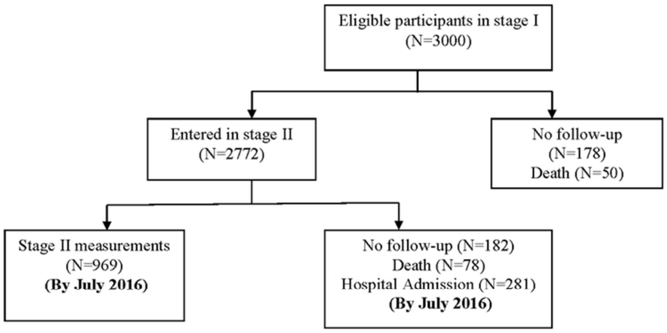

Figure1. Flow chart of enrolments Stage II

95x71mm (300 x 300 DPI)

For peer review only - http://bmjopen.bmj.com/site/about/guidelines.xhtml
